# Haematopoietic stem cell survival and transplantation efficacy is limited by the BH3-only proteins Bim and Bmf

**DOI:** 10.1002/emmm.201201235

**Published:** 2012-11-24

**Authors:** Verena Labi, Daniela Bertele, Claudia Woess, Denise Tischner, Florian J Bock, Sven Schwemmers, Heike L Pahl, Stephan Geley, Mirjam Kunze, Charlotte M Niemeyer, Andreas Villunger, Miriam Erlacher

**Affiliations:** 1 Division of Developmental Immunology, Biocenter, Innsbruck Medical UniversityInnsbruck, Austria; 2 Division of Pediatric Hematology and Oncology, Department of Pediatrics and Adolescent Medicine, University Hospital of FreiburgFreiburg, Germany; 3 Section of Molecular Hematology, Department of Hematology/Oncology, University Hospital of FreiburgFreiburg, Germany; 4 Division of Molecular Pathophysiology, Biocenter, Innsbruck Medical UniversityInnsbruck, Austria; 5 Department of Obstetrics and Gynecology, University Hospital FreiburgFreiburg, Germany; 6 Present address: Max Delbrück Center for Molecular Medicine (MDC)Berlin, Germany

**Keywords:** apoptosis, Bcl-2 protein family, Bmf, Bim, haematopoietic stem cell transplantation

## Abstract

Anti-apoptotic Bcl-2 family members are critical for the regulation of haematopoietic stem and progenitor cell (HSPC) survival. Little is known about the role of their pro-apoptotic antagonists, *i.e.* ‘BH3-only’ proteins, in this cell compartment. Based on the analysis of cytokine deprivation-induced changes in mRNA expression levels of Bcl-2 family proteins, we determined the consequences of BH3-only protein depletion on HSPC survival in culture and, for selected candidates, on engraftment *in vivo*. Thereby, we revealed a critical role for Bim and Bmf as regulators of HSPC dynamics both during early engraftment and long-term reconstitution. HSPCs derived from wild-type donors were readily displaced by Bim- or Bmf-deficient or Bcl-2-overexpressing HSPCs as early as 10 days after engraftment. Moreover, in the absence of Bim, significantly lower numbers of transplanted HSPCs were able to fully engraft radio-depleted recipients. Finally, we provide proof of principle that RNAi-based reduction of BIM or BMF, or overexpression of BCL-2 in human CD34^+^ cord blood cells may be an attractive therapeutic option to increase stem cell survival and transplantation efficacy.

## INTRODUCTION

At present, many haematological diseases can be cured only by transplantation of allogeneic haematopoietic stem and progenitor cells (HSPCs). Unfortunately, this treatment is still associated with a risk of graft failure, delayed engraftment or graft-*versus*-host disease. Clinical experience has shown that higher HSPC numbers result in faster haematopoietic regeneration, lowering the risk of graft failure (Demirer et al, 1996). Therefore, much effort is put in the development of new strategies to increase donor cell numbers based on stem cell mobilization, collection and *ex vivo* expansion (Rocha and Broxmeyer, 2010). An alternative approach to increase HSPC numbers might be the inhibition of cell death triggered by removal from the stem cell niche, subsequent transport, processing, storage and transplantation (de Boer et al, 2002; Greco et al, 2006; Schuurhuis et al, 2001). Extrusion of HSPCs from their niche is connected with a loss of pro-survival signals, mediated either by cytokines such as thrombopoietin (TPO), the c-Kit ligand stem cell factor (SCF) and vascular endothelial growth factor (VEGF), or by cell–cell contact (*e.g.* by delta-like-1 or Jagged1 mediated Notch-signalling) and cell–matrix contact (integrin α4β1/VLA) (Butler et al, 2010; Gerber et al, 2002; Murray et al, 1999; Qian et al, 2007; Varnum-Finney et al, 2000; Wang et al, 1998). Apoptosis in HSPCs in response to a lack of these signals has been studied but a precise molecular understanding of the signalling pathways involved is still lacking. Thus, whether inhibition of apoptosis induction is feasible and advantageous in haematopoietic stem cell transplantation (HSCT) regimens is still unclear.

It is well established that detachment of cells from the extracellular matrix or cytokine deprivation results in apoptosis mediated mainly through the intrinsic apoptosis pathway that is controlled by Bcl-2 family members (Cory et al, 2003). First evidence for an important role of Bcl-2-regulated apoptosis in HSPC homeostasis has been provided by the analysis of mice lacking or overexpressing different anti-apoptotic Bcl-2 proteins. Survival of HSPCs depends largely on Bcl-x_L_ and Mcl-1. Bcl-x_L_-deficient mice die around E13 and demonstrate extensive apoptosis of early haematopoietic cells in the foetal liver (Motoyama et al, 1995), and conditional depletion of Mcl-1 caused rapid depletion of HSPCs from bone marrow (BM) (Opferman et al, 2005). Of note, mice overexpressing Mcl-1 under the Vav-gene promoter developed lymphomas with a multipotent stem or progenitor cell phenotype at high frequency, and murine HSPCs overexpressing Mcl-1 showed increased colony forming potential (Campbell et al, 2010b). A recent publication suggests that Mcl-1 plays an important physiological function in human HSPCs as well (Campbell et al, 2010a). In contrast to Bcl-x_L_ and Mcl-1, loss of Bcl-2 does not overtly affect HSPC survival, and insufficient lymphocyte regeneration after serial transplantation of *bcl-2*^*−/−*^ BM cells has been proposed to be due to Bcl-2 dependence of lymphoid cells rather than HSPC defects (Matsuzaki et al, 1997; Veis et al, 1993). When overexpressed, however, transgenic Bcl-2 leads to an increased stem cell survival in the absence of c-Kit mediated signals (when expressed from the H2K promoter) as well as accumulation of HSPCs in foetal haematopoietic organs (Ly-6E/A promoter) or adult BM (H2K or Vav promoter). Moreover, Bcl-2 tg HSPCs resist a variety of chemotherapeutic agents and display enhanced clonogenic potential *in vitro* as well as an increased ability to reconstitute the haematopoietic system of lethally irradiated mice (Domen and Weissman, 2000, 2003; Domen et al, 1998, 2000; Ogilvy et al, 1999; Orelio et al, 2004).

While the role of different pro-survival Bcl-2 proteins appears well established, information on the relevance of their antagonists, the proteins of the BH3-only subgroup of the Bcl-2 family, including Bim, Bid, Puma and Bmf, is currently lacking. These proteins regulate the activation of Bax and Bak that ultimately perturb mitochondrial integrity, leading to apoptosis. As most BH3-only proteins show a redundant interaction pattern with different Bcl-2 pro-survival homologues (Chen et al, 2005), it currently remains unclear which BH3-only protein(s) regulate HSPC numbers under steady-state conditions or in response to transplantation stress. Detailed analysis of the relative contribution of individual BH3-only proteins on HSPC survival and clonogenic potential is lacking but seems warranted in the light of the broad range of applications involving HSPC transfer. In addition, since non-peptidic compounds that aim to mimic the pro-apoptotic function of BH3-only proteins are well-advanced in clinical trials as anti-cancer agents, the analysis of the physiological roles of BH3-only proteins in HSPCs is important to understand effects of these drugs on tissues with a high cellular turnover (Wilson et al, 2010). Hence, we characterized the expression pattern of BH3-only proteins in HSPCs and investigated their role in cytokine deprivation-mediated apoptosis *ex vivo* as well as in HSPC homeostasis under steady-state conditions *in vivo*. We also analysed the regeneration capacity of cells deficient for Bim or Bmf following stem cell transfer *in vivo.* Thereby, we demonstrate that both proteins limit early engraftment and long-term reconstitution of HSPCs in mice. Moreover, transplantation of HSPCs lacking Bim or Bmf significantly reduced the time required for successful host reconstitution. Finally, knockdown of these proteins in human cord blood-derived CD34^+^ cells enabled superior reconstitution of *rag2*^*−/−*^*γc*^*−/−*^ mice suggesting conserved functions of Bim and Bmf between humans and mice that may be exploited therapeutically to reduce HSCT-associated morbidity.

## RESULTS

### Multiple BH3-only proteins are induced in cytokine-deprived HSPCs but killing *in vitro* depends mainly on Bim

**Figure 1:**
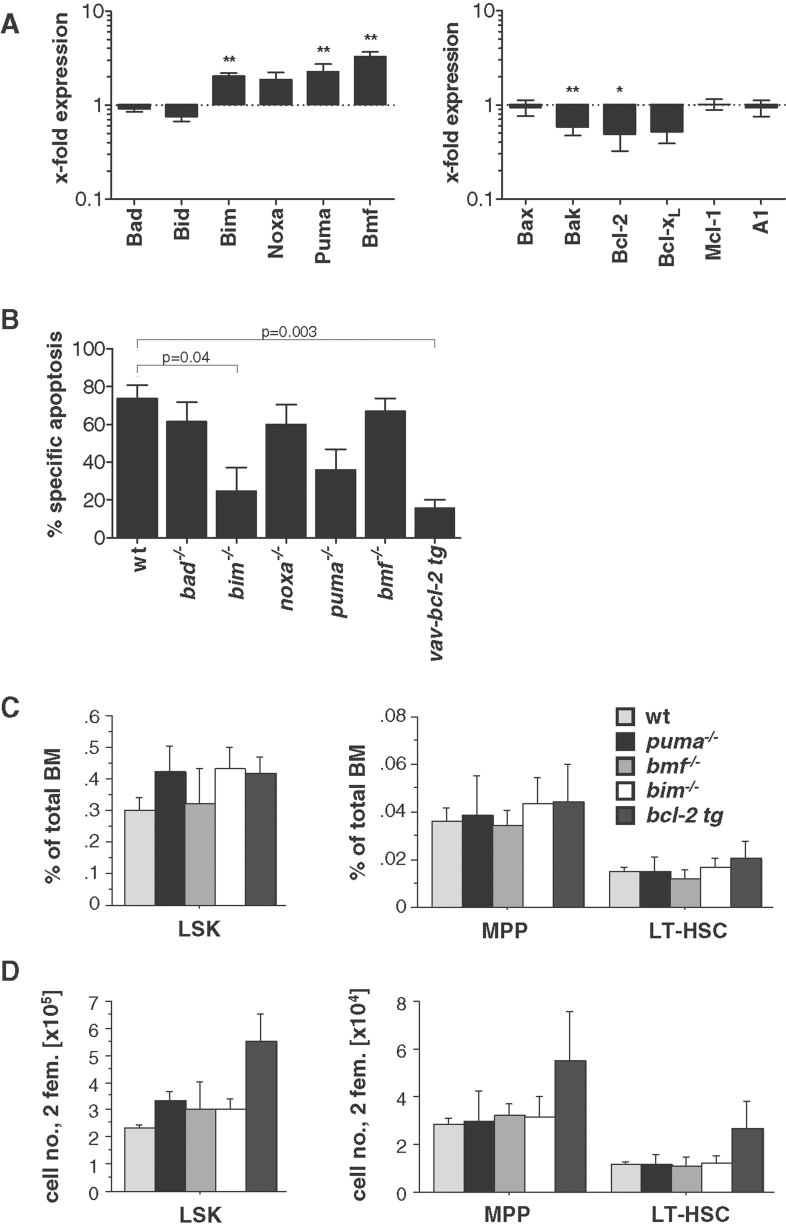
Bim- and Puma-mediated LSK cell killing upon growth factor deprivation Wt LSK cells were isolated from murine BM and cultured for 14 h in the presence or absence of SCF, TPO and Flt3L (100 ng/ml each). mRNA levels of the indicated BH3-only proteins, Bax and Bak as well as the anti-apoptotic Bcl-2 proteins were determined by RT-MLPA®. Blots show mRNA changes in the absence of cytokines when compared to mRNA levels in the presence of cytokines. Bars represent means of *n* = 5–6 from four independent experiments ± SEM. Significant *p* values: Bim *p* = 0.01; Puma *p* = 0.01; Bmf *p* = 0.01; Bak *p* = 0.01; Bcl-2 *p* = 0.04 (Mann–Whitney-test).LSK cells isolated from mice of the indicated genotypes were cultured for 48 h in the presence or absence of cytokines. Apoptosis was determined by combined staining with AnnexinV and 7-AAD, and specific apoptosis triggered by cytokine withdrawal was calculated by the following equation: (induced apoptosis − spontaneous apoptosis)/(100 – spontaneous apoptosis). Bars represent mean values of *n* = 3–4/genotype from three independent experiments ± SEM. Significant differences are indicated (Student *t*-test with Welch's correction).Eight- to ten-week-old mice of the indicated genotypes were sacrificed and BM was analysed by flow cytometry. Percentages of LSK as well as MPP and LT-HSC were determined by cell surface staining.Total numbers of LSK, MPP and LT-HSC were determined by multiplying BM cellularity (2 femurs) by the indicated percentages. Bars represent means from three to four mice/genotype from two independent experiments ± SEM. No significant differences were observed (Mann–Whitney-test).

To analyse which BH3-only proteins are induced in the absence of cytokine-mediated survival signals and may therefore mediate HSPC apoptosis, we isolated LSK cells from BM of wt mice and cultured them in the presence or absence of the cytokines TPO, Flt3L and SCF. Employing RT-MLPA® analysis allowed us to screen relative changes in the mRNA expression of all known Bcl-2 family members as well as different additional apoptosis-related genes (Eldering et al, 2003). We observed cytokine deprivation-induced changes in the mRNA levels of Bim (2.0-fold), Bmf (3.3-fold), Puma (2.3-fold) and Noxa (1.9-fold), but not Bad, Bid (Fig 1A) or Bik (not expressed). Induction of mRNA levels was independently confirmed by qRT-PCR (Supporting Information Fig 1A). No upregulation of Bax or Bak was observed whereas their pro-survival antagonists Bcl-2 and Bcl-x_L_ were downregulated at the mRNA level (0.5-fold, both; Fig 1A and Supporting Information Fig 1A). The levels of Mcl-1 or A1 mRNA (Fig 1A) as well as of all remaining genes monitored were found to be largely unchanged (Supporting Information Table II).

To test the relevance of these expression changes for cytokine deprivation-induced apoptosis, we isolated LSK cells from mice lacking Bim, Puma, Noxa, Bmf or Bad. Surprisingly, only loss of Bim and, to a lesser extent, Puma led to a protection of LSK cells from cytokine deprivation-induced apoptosis *in vitro*, whereas loss of Bmf, Noxa or Bad did not prevent apoptosis (Fig 1B). As published previously, LSK cells overexpressing Bcl-2 under control of the *Vav*-gene promoter were almost completely resistant to cytokine deprivation-induced apoptosis (Fig 1B) (Ogilvy et al, 1999; Domen and Weissman, 2000).

Next, we analysed the HSPC compartment of age and sex-matched wt, *bim*^*−/−*^, *puma*^*−/−*^, *bmf*^*−/−*^ and *vav-bcl-2 tg* mice. No differences in the percentages of LSK cells, multi-potent progenitors (MPP: LSK CD48^−^CD150^−^) or in a compartment highly enriched for long-term repopulating stem cells (LSK CD48^−^CD150^+^, referred to as long-term HSC; LT-HSC) were observed between the genotypes (Fig 1C). Similarly, total cell numbers of LSK, MPP and LT-HSC were comparable between genotypes with the exception of *vav-bcl-2 tg* mice, due to their mildly increased total BM cellularity (Fig 1D). Thus, none of the BH3-only proteins analysed is rate-limiting for HSPC homeostasis under steady-state conditions on its own. Our data hence suggests that some of these proteins act redundantly in HSPC homeostasis.

### Efficiency of long-term haematopoietic reconstitution is increased in the absence of Bim

**Figure 2:**
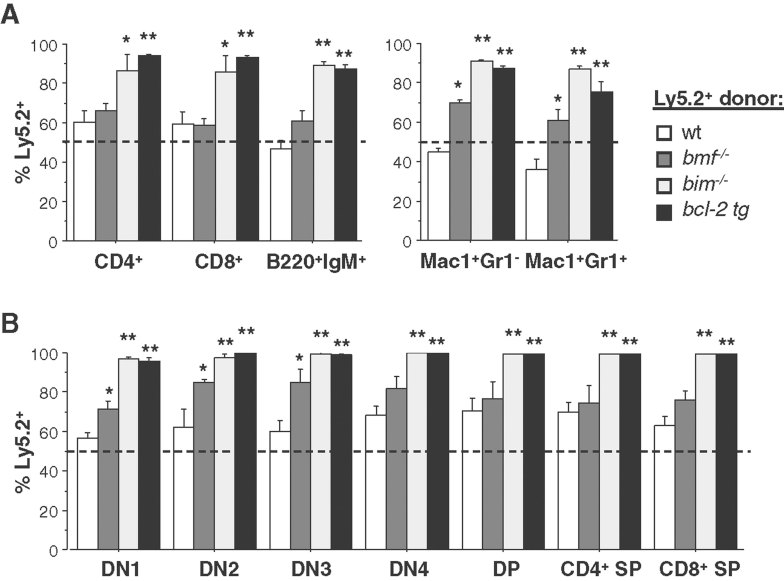
Displacement of wt haematopoiesis by *bim^−/−^* or *bcl-2 tg* cells **A,B.** 16 weeks after competitive transplantation recipient mice were sacrificed, and (**A**) spleens and (**B**) thymi were analysed in detail by flow cytometric analysis. Surface markers identifying immature thymocyte subsets, T, B and myeloid cells were combined with antibodies against Ly5.1 and Ly5.2 (DN: CD4^−^8^−^; DN1: CD44^+^25^−^; DN2: CD44^+^25^+^; DN3: CD44^−^25^+^; DN4: CD44^−^CD25^−^). Bars represent means of *n* = 4–6 animals per genotype from four independent experiments ± SEM. Significant *p* values (Mann–Whitney-test): wt *versus bmf*^*−/−*^: *p* = 0.03 in monocytes and DN3 thymocytes; *p* = 0.05 in granulocytes, DN1 and DN2 thymocytes. Wt *versus bim*^*−/−*^: *p* = 0.01 in B cells, myeloid cells and thymocytes; *p* = 0.03 in CD4^+^ and CD8^+^ cells. Wt *versus bcl-2 tg* cells: *p* = 0.01 in all cell types. No significant differences between *bim*^*−/−*^ and *bcl-2 tg* cells were obtained.

We next asked whether the prolonged survival of *bim*^*−/−*^ LSK cells under suboptimal conditions *in vitro* would translate into a better reconstitution of lethally irradiated mice. We also included *bmf*^*−/−*^ LSK cells in our *in vivo* studies because Bmf mRNA was induced strongest after cytokine deprivation. To directly compare the reconstitution ability of *bim*^*−/−*^ or *bmf*^*−/−*^ to that of wt LSK cells, we performed competitive reconstitution experiments. Lethally irradiated Ly5.1^+^ recipients were reconstituted with a 50:50 mixture of LSK cells derived from a Ly5.1^+^ wt and a Ly5.2^+^ wt, *bim*^*−/−*^, *bmf*^*−/−*^ or *vav-bcl-2 tg* mouse. After 16 weeks, recipient mice were sacrificed and haematological organs analysed. Mature splenic T and B cells of wt:wt BM chimeras displayed nearly the expected 50:50 ratio (Ly5.1^+^:Ly5.2^+^ = 44:56%) between the two donors (Fig 2A and Supporting Information Fig 2). In contrast, in wt:*bim*^*−/−*^ chimeras almost all splenic T and B cells were derived from the Ly5.2^+^ *bim*^*−/−*^ donor. Of note, chimeras that received BM from *vav-bcl-2 tg* mice were not significantly different from those that received *bim*^*−/−*^ cells, implicating that Bim is the major rate limiting BH3-only protein during reconstitution and accounts for all apoptotic cell death blocked by Bcl-2 overexpression (Fig 2A).

Bim has previously been described to play an important role in various lymphocyte selection processes (reviewed by Strasser, 2005). The displacement of mature wt lymphocytes by *bim*^*−/−*^ or *bcl-2* tg cells observed in our competitive reconstitution experiments may, therefore, simply mirror the cumulative survival of apoptosis-resistant lymphocytes throughout development. However, the biased ratio of 13%:87% (Ly5.1^+^:Ly5.2^+^) seen in mature splenic CD4^+^ T cells remained stable throughout all developmental stages in the T-lineage analysed (Fig 2B). Similarly, most immature B cells (splenic T1 and T2 cells and BM-derived B220^+^IgM^−^ cells) were of *bim*^*−/−*^ origin (Table 1, Supporting Information Table III). The effects observed in wt:*bmf*^*−/−*^ chimeras, while statistically significant, were less pronounced and cell type-dependent (Fig. 2, Table 1 and Supporting Information Table III).

**Table 1:** Ly5.2^+^/Ly5.1^+^ ratio in wt:wt, wt:*bmf*^*−/−*^, wt:*bim*^*−/−*^ and wt:*bcl-2 tg* BM chimeras

	wt:wt	wt:*bmf*^*−/−*^	*p* value	wt:*bim*^*−/−*^	*p* value	wt:*bcl-2tg*	*p* value
Bone marrow							
LSK	1.12 ± 0.08	2.57 ± 0.34	0.03	11.47 ± 2.65	0.01	10.12 ± 2.85	0.01
LSKCD150^+^	1.06 ± 0.12	3.71 ± 1.12	0.03	6.69 ± 1.27	0.01	7.31 ± 0.70	0.01
preB cells	1.58 ± 0.25	2.69 ± 0.10	0.03	19.62 ± 2.86	0.01	63.63 ± 24.29	0.01
Monocytes	1.28 ± 0.16	4.51 ± 1.74	0.03	15.36 ± 2.72	0.01	8.46 ± 0.99	0.01
Granulocytes	1.45 ± 0.15	4.60 ± 2.014	0.03	12.95 ± 1.48	0.01	6.66 ± 0.70	0.01
Thymus							
DN	1.48 ± 0.19	3.50 ± 1.42	0.10	169.06 ± 36.7	0.01	308.44 ± 56.8	0.01
DP	3 55 ± 1 47	6 47 ± 4 27	0 65	259 22 ± 66 3	0 01	157 37 ± 86 9	0 01
CD4^+^SP	2.85 ± 0.83	4.68 ± 2.63	0.65	219.45 ± 49.7	0.01	159.64 ± 18.8	0.01
CD8^+^SP	1.91 ± 0.45	3.56 ± 1.01	0.10	319.89 ± 78.3	0.01	237.25 ± 98.9	0.01
Spleen							
CD4^+^	2.78 ± 1.25	2.61 ± 0.47	0.30	24.44 ± 7.89	0.03	31.26 ± 3.86	0.01
CD8^+^	2.15 ± 0.86	1.63 ± 0.18	0.65	15.84 ± 4.86	0.03	21.05 ± 3.97	0.01
B220^+^IgM^+^	1.37 ± 0.14	3.63 ± 0.64	0.03	31.34 ± 10.99	0.01	19.02 ± 4.23	0.01
T1 B cells	1.70 ± 0.11	4.79 ± 0.78	0.03	61.94 ± 28.48	0.01	81.87 ± 19.47	0.01
T2 B cells	1.97 ± 0.15	2.87 ± 0.19	0.03	22.79 ± 3.00	0.01	53.11 ± 33.10	0.01
Monocytes	0.91 ± 0.09	2.56 ± 0.15	0.03	12.71 ± 1.20	0.01	8.43 ± 0.70	0.01
Granulozytes	0.80 ± 0.16	3.15 ± 0.41	0.03	86.48 ± 72.28	0.01	35.85 ± 31.09	0.01
Ter119^+^	0.99 ± 0.29	2.47 ± 0.87	0.18	49.38 ± 9.98	0.01	38.61 ± 8.43	0.01
Blood							
CD4^+^	1.64 ± 0.14	2.80 ± 0.45	0.03	17.47 ± 3.75	0.01	18.43 ± 5.07	0.01
CD8^+^	1.04 ± 0.10	1.37 ± 0.06	0.05	15.08 ± 0.41	0.01	10.97 ± 4.16	0.01
B220^+^IgM^+^	1.54 ± 0.27	5.53 ± 1.04	0.03	19.52 ± 11.64	0.03	15.63 ± 7.44	0.03
Monocytes	0.88 ± 0.12	2.14 ± 0.27	0.03	11.05 ± 1.26	0.01	5.31 ± 1.71	0.01
Granulocytes	1.03 ± 0.13	1.72 ± 0.29	0.10	5.95 ± 1.04	0.01	3.47 ± 0.66	0.01

Sixteen weeks after competitive transplantation recipient mice were sacrificed, and all indicated haematopoietic organs were analysed in detail by flow cytometry using the indicated cell surface marker-specific antibodies (exemplary dot plots are shown in Supporting Information Fig 2). An almost complete and highly significant displacement of wt Ly5.1^+^ cells can be observed in wt:*bim*^*−/−*^ and wt:*bcl-2 tg* chimeras. Bmf-deficiency could only partially replace wt haematopoiesis. Values represent means of *n* = 4–6 animals per genotype from four independent experiments ± SEM. All *p* values are indicated (Mann–Whitney-test).

**Figure 3:**
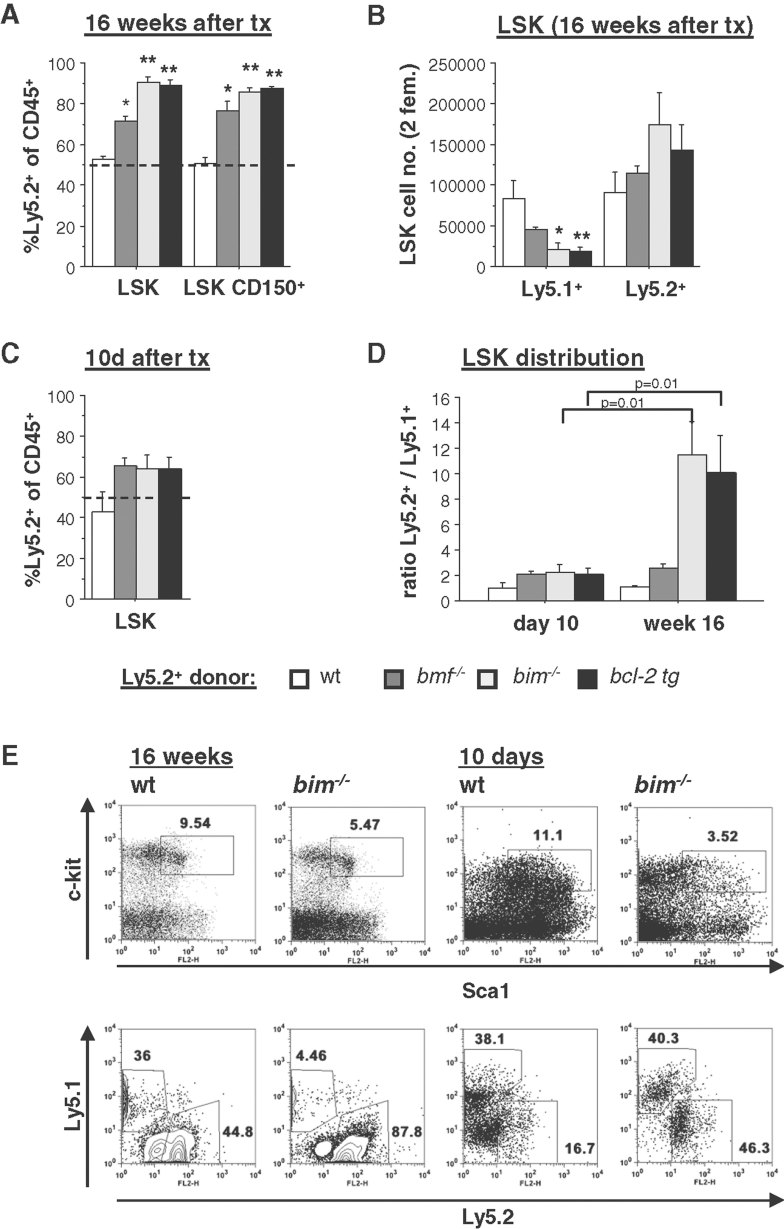
Displacement of wt haematopoiesis in wt:*bmf*^−/−^, wt:*bim* *^−/−^* and wt:*bcl-2* tg BM chimeras starts within the HSPC compartment Sixteen weeks after competitive reconstitution, recipient mice were sacrificed and the HSPC compartment was analysed by flow cytometry to determine percentages of Ly5.2^+^ LSK as well as LSK CD150^+^ cells. Significant *p* values (Mann–Whitney-test): wt *versus bmf*^*−/−*^: *p* = 0.03 in LSK and LSK CD150^+^ cells, wt *versus bim*^*−/−*^ and wt *versus bcl-2 tg* cells: *p* = 0.01 in LSK and LSK CD150^+^ cells.Total numbers of Ly5.1^+^ and Ly5.2^+^ LSK cells depict the almost complete displacement of wt LSK cells in wt:*bim*^*−/−*^ and wt:*bcl-2 tg* chimeras. Significant *p* values (Mann–Whitney-test): wt *versus bim*^*−/−*^: *p* = 0.03 in Ly5.1^+^ cells; wt *versus bcl-2 tg* cells: *p* = 0.01 in Ly5.1^+^ cells.Similarly, %Ly5.2^+^ LSK cells were determined 10 days after competitive reconstitution.The ratio Ly5.2^+^/Ly5.1^+^ cells within the LSK compartment changes significantly between Day 10 and week 16 in wt:*bim*^*−/−*^ and wt:*bcl-2 tg* chimeras. Bars represent means of three to six animals/genotype from three independent experiments ± SEM.Representative dot plots of LSK cells derived from wt and *bim*^*−/−*^ mice 10 days and 16 weeks after transplantation are shown.

Analysis of the erythro-myeloid lineage revealed that monocytes, granulocytes and erythroid progenitors were also derived to a great extent from the *bim*^*−/−*^, *bmf*^*−/−*^ or *bcl-2* tg donors 16 weeks after transplantation (Fig 2A, Supporting Information Fig 2, Table 1 and Supporting Information Table 3). Since granulocytes are short-lived cells (Pillay et al, 2010), they best mirror the composition of HSPCs at any given time point. Therefore, we concluded that *bim*^*−/−*^ or *bcl-2* tg cells account for the majority of HSPCs in BM chimeras. Indeed, around 90% of LSK or the more immature LSK CD150^+^ cells were derived from the Bim-deficient donor in wt:*bim*^*−/−*^ chimeras 16 weeks after reconstitution (Fig 3A, B and E, Table 1 and Supporting Information Table III). In line with its strong mRNA induction after cytokine deprivation, but in contrast to the lack of cytoprotection upon cytokine deprivation *ex vivo* (Fig 1A and B), loss of Bmf also led to a significant displacement of Ly5.1^+^ wt LSK cells in reconstituted mice (Fig 3A and B). These data demonstrate that *bim*^*−/−*^, *bmf*^*−/−*^ and *bcl-2* tg LSK cells harbour a significant competitive advantage in BM reconstitution experiments and that wt haematopoiesis is suppressed or displaced completely by *bim*^*−/−*^ and *bcl-2* tg cells and to a significant degree also by *bmf*^*−/−*^ cells. Importantly, no lymphoma or leukaemias occurred in recipient mice that were monitored in parallel over an observation period of 8 months (wt donor: *n* = 13, *bim*^*−/−*^ donor: *n* = 14; *bmf*^*−/−*^ donor: *n* = 10).

### Bim- and Bmf-dependent killing limits the reconstitution potential of HSPCs *in vivo*

We wondered whether HSPCs deficient for Bim or Bmf performed better than wt HSPCs from the very beginning or whether these cells accumulated over time. We thus analysed recipient mice already 10 days after competitive transplantation. Surprisingly, even at this early time point a clear difference in the ratio between Ly5.1^+^ and Ly5.2^+^ LSK cells was observed in BH3-only protein defective or Bcl-2 overexpressing chimeras (Fig 3C–E). BM analysis at earlier time points was complicated by highly variable responses, in part due to large numbers of dying recipient BM cells and low numbers of donor-derived LSK cells detectable by flow cytometry, but consistently showed a trend that early engraftment was facilitated by loss of Bim or overexpression of Bcl-2 (Supporting Information Fig 1C). Next, we asked whether mechanisms other than apoptosis inhibition could contribute to the superior performance of *bim*^*−/−*^ HSPC. In both homing and proliferation experiments, however, no difference between wt and *bim*^*−/−*^ LSK cells was observed (Supporting Information Fig 3). Importantly, proliferative capacity was assessed prior transplantation, during stress haematopoiesis (4 weeks after transplantation) and, in order to test cell cycle progression in response to physiological cues, after 3 days of culture in the presence of cytokines (Supporting Information Fig 3). Altogether, these data provide compelling evidence that during transplantation, apoptosis of wt cells contributes to the loss of HSPCs.

**Figure 4:**
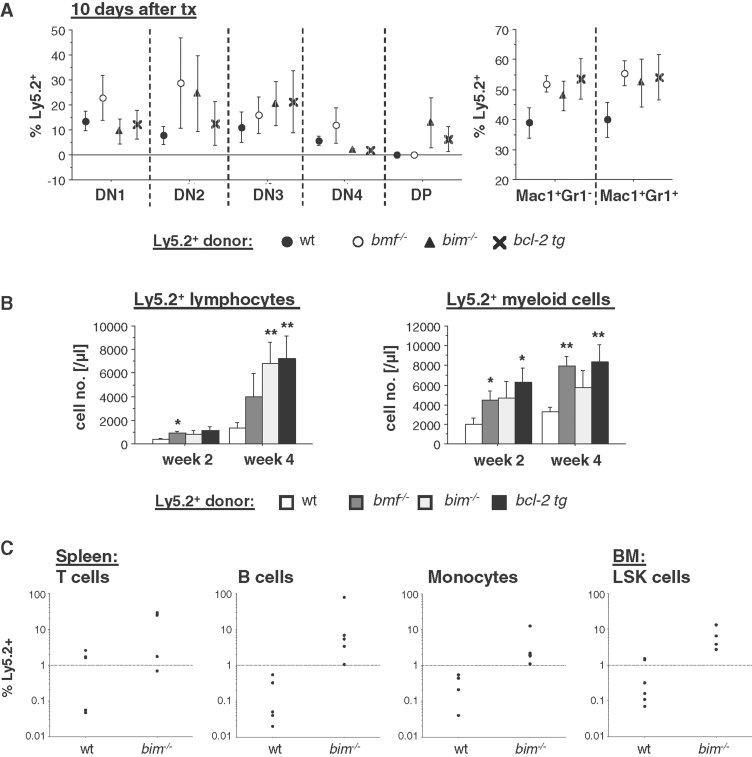
Bim- and Bmf-dependent apoptosis limits the reconstitution potential of transplanted HSPC *in vivo* Ten days after competitive reconstitution, recipient mice were sacrificed and percentages of Ly5.2^+^ thymocytes were determined by flow cytometry. Thymocyte subsets were defined by surface expression of CD4, CD8, CD25 and CD44. Similarly, percentages of Ly5.2^+^ Mac1^+^Gr1^−^ monocytes and Gr1^+^ granulocytes in the spleen were determined 10 days after transplantation. Symbols represent %Ly5.2^+^ cells within the indicated cell populations.Two and 4 weeks after competitive reconstitution, blood samples were collected from recipient mice and white blood cells were stained with antibodies against CD4, CD8, B220, Gr1, Mac1, Ly5.1 and Ly5.2. Bars represent absolute numbers of Ly5.2^+^ lymphocytes (CD4^+^ and CD8^+^ T cells plus B220^+^ B cells) and Ly5.2^+^ myeloid cells (monocytes plus granulocytes). Symbols represent means of four to five mice/genotype from three independent experiments ± SEM. Significant *p* values (Mann–Whitney-test): wt *versus bmf*^*−/−*^: *p* = 0.05 in lymphocytes (2 weeks), *p* = 0.03 in myeloid cells (2 weeks) and *p* = 0.01 in myeloid cells (4 weeks). Wt *versus bim*^*−/−*^: *p* = 0.01 in lymphocytes (4 weeks). Wt *versus bcl-2 tg* cells: *p* = 0.01 in lymphocytes and myeloid cells (4 weeks), and *p* = 0.03 in myeloid cells (2 weeks).Lethally irradiated recipient mice were transplanted with 10,000 total BM cells derived from Ly5.2^+^ wt or *bim*^*−/−*^ mice. Two-hundred thousand Ly5.1^+^ wt BM cells were used as competitor cells. Fourteen weeks after transplantation, %Ly5.2^+^ cells were determined in lymphatic and myeloid cell subsets and successful engraftment was defined by >1% Ly5.2^+^ myeloid and >1% Ly5.2+ lymphatic splenic cells. The right panel shows %Ly5.2^+^ cells of BM resident LSK cells. Differences in engraftment were determined with the Fisher exact test (*p* = 0.015).

To define the relevance of the early reconstitution advantage of *bim*^*−/−*^ or *bmf*^*−/−*^ LSK cells for the kinetics of haematopoietic regeneration, we analysed BM, spleen and thymus at 5 and 10 days after reconstitution. At day 5, all haematopoietic organs were reduced in size as well as cellularity and almost no differentiated Ly5.2^+^ cells were detectable, independent of the donor-genotype (unpublished observation). Similarly, at Day 10 most of the cells were host-derived. At this time point, Ly5.2^+^ and thus LSK-derived donor cells were detectable up to the DN3-4 stage and a trend to higher percentages of Ly5.2^+^ cells was already observed in wt:*bim*^*−/−*^, wt:*bmf*^*−/−*^ and wt:*bcl-2* tg chimeras when compared to wt:wt chimeras. The percentages of splenic Ly5.2^+^ monocytes and granulocytes were likewise increased in the absence of Bim or Bmf (Fig 4A). In the peripheral blood, significant advantages of *bim*^*−/−*^, *bmf*^*−/−*^ and *bcl-2 tg* cells were first noted 2 and 4 weeks after transplantation and affected both lymphocytes and myeloid cells (Fig 4B). Of note, whereas *bmf*^*−/−*^ cells were clearly less potent in replacing wt cells in long-term reconstitution experiments, when compared to *bim*^*−/−*^ cells, they appeared equally potent during the initial haematopoietic reconstitution (Fig 4A and B). This observation is in line with our hypothesis that both, increased stem cell survival as well as accumulation of mature lymphatic and myeloid cells, contribute to the phenotypes observed in our long-term assays. Hence, the role of Bmf may be more critical during initial reconstitution events.

Because our data suggested that the reconstitution potential of wt HSPCs is limited by Bim-dependent apoptosis under conditions of reconstitution stress, we transplanted rate limiting numbers of wt or *bim*^*−/−*^ BM-derived cells simultaneously with 200,000 Ly5.1^+^ BM competitors. Successful transplantation was defined by long-term engraftment >1% of both the myeloid and the lymphatic lineage (Akala et al, 2008). Indeed, 10,000 *bim*^*−/−*^ BM cells were still able to successfully engraft recipient mice whereas wt donor cells failed to give >1% engraftment (*n* = 4/5 for *bim*^*−/−*^ donors, *n* = 0/6 for wt donors, *p* = 0.015 in Fisher exact test; Fig 4C).

### HSPC aging is not influenced by loss of Bim or Bmf or overexpression of Bcl-2

To investigate long-term repopulating activity, self-renewal and aging of *bim*^*−/−*^, *bmf*^*−/−*^ and *vav-bcl-2 tg* HSPC, we performed serial transplantations at 16-week intervals. To increase competitive pressure during secondary and tertiary transplantation, 5000 Ly5.2^+^ LSK cells were transplanted along with 200,000 total Ly5.1^+^ BM cells from unchallenged donors. Whereas Ly5.2^+^ LSK cells always successfully reconstituted secondary recipients under these conditions, no Ly5.2^+^ engraftment was detected following tertiary transplantation, independently of the donor genotype (Supporting Information Fig 4). Importantly, over the whole time course of this experiment no cell death deficiency-related pathology, such as autoimmune diseases or lymphoma occurred in recipient mice.

### Conserved function of BIM and BMF in human HSPC survival

**Figure 5:**
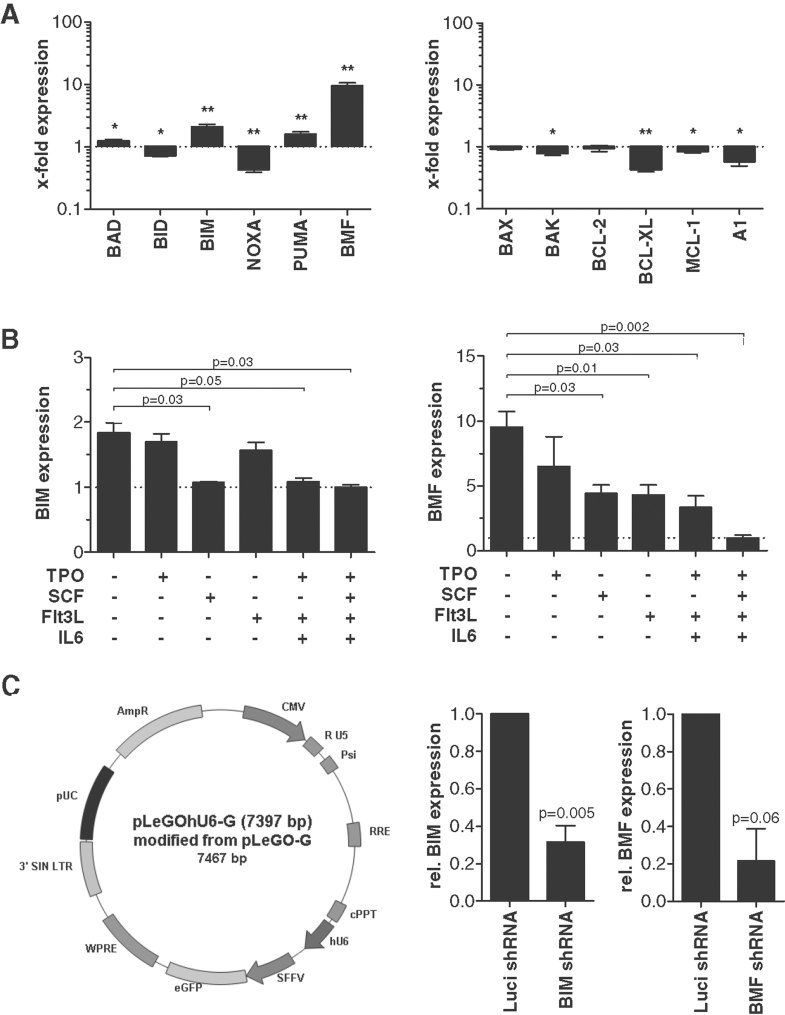
Factor deprivation triggers induction of BIM and BMF in human CD34^+^ cells Fresh cord blood derived CD34^+^ cells were cultured in the presence or absence of SCF, Flt3L, IL6 (100 ng/ml each) and TPO (10 ng/ml). After 14 h of cytokine deprivation, mRNA levels of the indicated BH3-only proteins, BAX and BAK as well as the anti-apoptotic BCL-2 proteins were determined by RT-MLPA®. Blots show mRNA changes in the absence of cytokines when compared to mRNA levels in the presence of cytokines (*n* = 7, 5 independent experiments). Significant *p* values (Mann–Whitney-test): BAD *p* = 0.05; BID *p* = 0.02; BIM *p* = 0.009; NOXA *p* = 0.002; PUMA *p* = 0.009; BMF *p* = 0.002; BAK *p* = 0.05; BCL-XL *p* = 0.002; MCL-1 *p* = 0.05; A1 *p* = 0.03.Addition of single cytokines or combinations thereof led to differential downregulation of BIM and BMF when compared to mRNA expression in the absence of cytokines (1 = mRNA expression in the presence of SCF, Flt3L, TPO and IL-6). Bars represent means of 3–7 from three independent experiments ± SD. Significant *p* values (Mann–Whitney-test) are indicated.Lentiviruses were constructed by using the pLeGOhU6-G vector, as published by Roelz et al. Transduction efficacy in CD34^+^ cells was monitored by eGFP expression and generally was 70–80%. qRT-PCR showed that shRNA led to an efficient downregulation of either BIM or BMF in GFP^+^CD34^+^ cells. Bars show mean mRNA expression of *n* = 3–5 per shRNA from three independent experiments ± SD. Significant *p* values (Mann–Whitney-test) are indicated.

To explore the possibility of a beneficial effect of apoptosis modulation for HSCT in humans, we isolated fresh cord blood-derived CD34^+^ cells, a cell population enriched for HSPCs and used as a source of HSCT grafts in certain transplantation regimens such as haplo-identical transplantations. Subjecting these cells to cytokine withdrawal *in vitro* revealed that human CD34^+^ cells were generally less susceptible to cytokine deprivation-induced apoptosis than murine LSK cells (comp. Fig 1B and Supporting Information Fig 5A). RT-MLPA analysis performed 14 h after cytokine withdrawal indicated a more restricted upregulation of BH3-only proteins with significant induction noted only for BIM and BMF mRNA (2.1- and 9.5-fold, respectively) and only minor induction of PUMA mRNA (1.6-fold), an observation again confirmed by qRT-PCR (Fig 5A and Supporting Information Fig 5B).

Concomitantly, mRNA encoding for the anti-apoptotic protein BCL-XL was significantly downregulated while levels of BAX or BAK remained unchanged (Fig 5A). Regulation of other genes monitored in RT-MLPA analysis revealed no or only minor changes with the notable exception of Birc1, Bnip3L and Survivin (Supporting Information Table II), the latter implicated in cell death as well as cell cycle regulation of HSPCs (Fukuda and Pelus, 2002). BIM induction was efficiently repressed by SCF, while Flt3L or TPO alone had no effect. In contrast, BMF induction was partially repressed either by SCF or Flt3L (Fig 5B).

**Figure 6:**
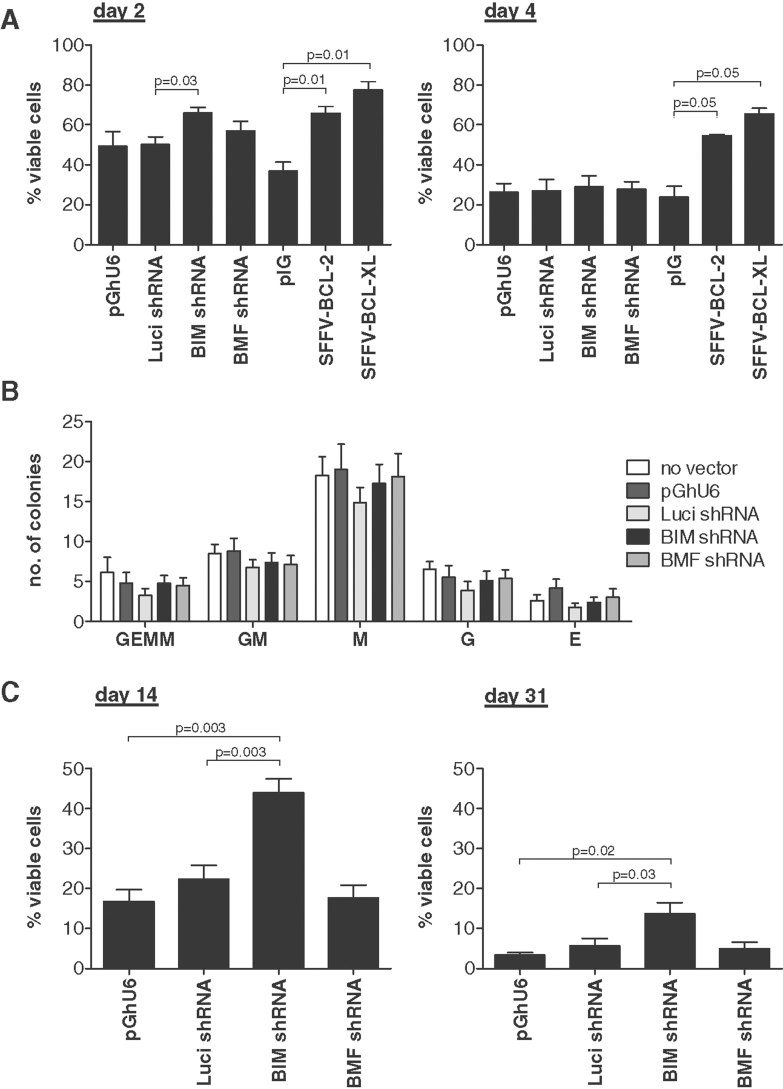
Downregulation of BIM but not BMF leads to survival advantages of CD34^+^ cells *in vitro* CD34^+^ cells were transduced with the indicated lentiviral particles. GFP^+^ cells were cultured for 2 or 4 days in the absence of cytokines, stained with AnnexinV and 7-AAD and analysed by flow cytometry. Bars show a mean of *n* = 4–5 from three independent experiments ± SEM. Significant *p* values (Mann–Whitney-test) are indicated.CD34^+^ cells transduced with the indicated lentiviral particles were cultured in Methocult medium supplemented with G-CSF, GM-CSF, IL-3, IL-6 and EPO. On Day 11 of culture, colonies were analysed by light microscopy and quantified based on typical morphological features (mixed: GEMM, myeloid: GM + M + G, erythroid: E). Bars represent means of *n* = 6–8 from five independent experiments ± SEM; no significant differences were observed (Mann–Whitney-test).Fourteen days following plating, cells were harvested from Methocult plates and viability was determined by combined staining with AnnexinV and 7-AAD (left panel). Part of the cells was replated in fresh Methocult plates, harvested 17 days later and analysed accordingly to the first plating (right panel). Bars represent means of *n* = 5–8 from five independent experiments (left) or *n* = 3–6 from three independent experiments (right) ± SEM, significant *p* values are indicated (Mann–Whitney-test).

Lentivirally transduced shRNA specific for BIM or BMF was used to test the relevance of these two BH3-only proteins for the induction of human HSPC apoptosis. In addition, lentiviruses were generated for expression of their antagonists BCL-2 and BCL-XL. The RNAi-knockdown efficiency was determined by qRT-PCR for BIM and BMF mRNA (Fig 5C) and by immunoblotting for target proteins isolated from sorted GFP^+^ CD34^+^ cells (Supporting Information Fig 5C). RNA levels of BIM and BMF were reduced by ∼69 and ∼78%, respectively, at the time of analysis (Fig 5C). Culturing GFP^+^ CD34^+^ cells for 2 days in the absence of cytokines revealed that knockdown of BIM but not BMF delayed cytokine deprivation-induced apoptosis *ex vivo*, although this effect was not as pronounced as that seen in Bim-deficient mouse HSPCs (comp. Figs 6A, 1B). After 4 days BIM knockdown proved inefficient, whereas overexpression of BCL-2 or BCL-XL still effectively increased survival (Fig 6A, right panel).

In order to determine the impact of BIM or BMF downregulation on HSPC differentiation and growth, we performed colony-forming assays (Methocult®). No differences in colony formation and colony size were observed between the different groups after 11 days of culture (Fig 6B). However, on day 14, colonies derived from CD34^+^ cells expressing BIM shRNA were larger and contained more viable cells. Increased survival of BIM shRNA expressing progenitors was likewise observed following replating on Day 31 (Fig 6C). BMF shRNA, in contrast, was not able to inhibit CD34^+^ cell apoptosis induced in the presence or absence of cytokines (Fig 6A and C).

**Figure 7:**
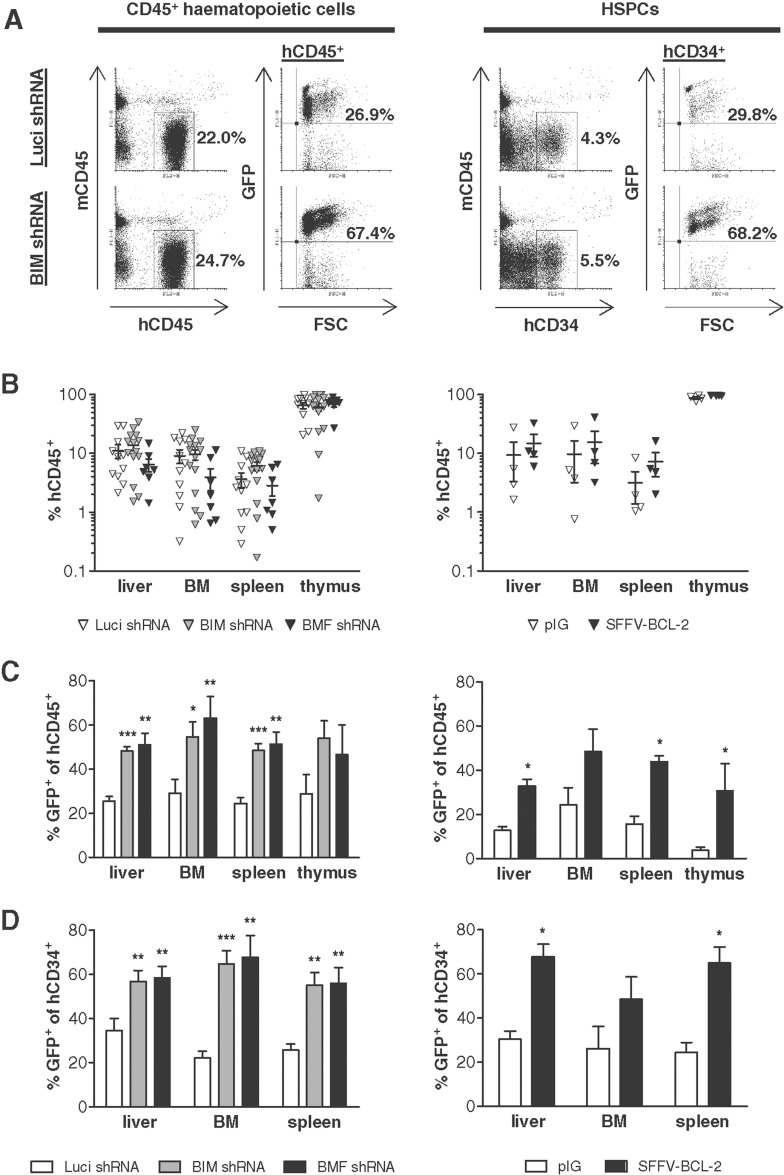
Knockdown of BIM or BMF improves engraftment of human CD34^+^ cells **A.** Cord blood derived human CD34^+^ cells were transduced with the indicated lentiviruses and injected intrahepatically into newborn *rag2*^*−/−*^*γc*^*−/−*^ mice. Eight weeks later, animals were sacrificed. Single cell suspensions were stained with antibodies against human CD45 (left) or CD34 (right) and murine CD45, and the percentages of GFP^+^ cells within the human populations were determined. Dot blots of representative experiments are shown.**B.** Eight weeks after transplantation, the indicated haematological organs were analysed. Total human engraftment was determined by staining single cell suspensions with antibodies against human and murine CD45. Lentiviruses expressed shRNA specific for Luciferase, BIM or BMF (left panel) or overexpressed BCL-2 under control of the SFFV promoter (right panel). No significant differences in total human engraftment could be observed between the different groups (*n* = 7–14 from four to six independent experiments, left panel; and *n* = 4 from two independent experiments, right panel; Mann–Whitney-test).**C,D.** The percentage of GFP^+^ cells within the human CD45^+^ (**C**) or the immature CD34^+^ (**D**) cell population was determined by flow cytometry. Knockdown of BIM or BMF (left panel) as well as overexpression of BCL-2 (right panel) clearly increased percentages of GFP^+^ cells. Significant *p* values (Mann–Whitney-test): %GFP^+^ of hCD45^+^ cells: Luci shRNA *versus* BIM shRNA: liver *p* < 0.0001, BM *p* = 0.02, spleen *p* = 0.0002; Luci shRNA *versus* BMF shRNA: liver *p* = 0.002, BM *p* = 0.009, spleen *p* = 0.004; pIG *versus* BCL-2 *p* = 0.03 in liver, spleen and thymus. %GFP^+^ of hCD34^+^ cells: Luci shRNA *versus* BIM shRNA: liver *p* = 0.006, BM *p* = 0.0001, spleen *p* = 0.001; Luci shRNA *versus* BMF shRNA: liver *p* = 0.009, BM *p* = 0.002, spleen 0.005; pIG *versus* BCL-2: *p* = 0.03 in liver and spleen.

To investigate the potency of human HSPCs with reduced levels of BIM or BMF to engraft and to compete against non-manipulated HSPCs we used newborn *rag2*^*−/−*^*γc*^*−/−*^ mice and injected them intrahepatically with the progeny of 10^5^ CD34^+^ cells transduced with lentiviruses either expressing shRNAs targeting Luciferase, BIM or BMF mRNA or a BCL-2 transgene. Transduction efficiencies were monitored before transplantation and found comparable between the vectors (Supporting Information Fig 6A). Eight weeks after transplantation overall human engraftment, defined by percentages of human CD45^+^ cells was similar in all groups analysed (Fig 7A and B). However, in all analysed organs a clearly higher proportion of GFP^+^ cells was detected when CD34^+^ donor cells expressed BIM or BMF shRNA, corroborating our results obtained in the murine *in vivo* system (Fig 7A and C). Notably, significant competitive advantages of GFP^+^ cells expressing BIM or BMF shRNAs could not only be observed in differentiated CD19^+^ B cells and CD33^+^ myeloid cells (Supporting Information Fig 6B and C) but was already visible in immature human CD34^+^ cells isolated from murine livers, BM and spleens (Fig 7A and D). The effects obtained by BCL-2 overexpression were similar (Fig 7B–D, right panels) indicating that BIM and BMF account for the majority of apoptosis induction during transplantation of CD34^+^ cells. Since intrahepatic transplantation may only partially mimic intraosseous engraftment occurring during clinical HSCT, we additionally injected human CD34^+^ cells intravenously into adult recipient mice. Results were comparable with those obtained in newborn mice with a clear engraftment advantage of CD34^+^ cells expressing BIM shRNA in all cell subsets analysed (Supporting Information Fig 7).

## DISCUSSION

Graft failure and delayed haematopoietic and immune reconstitution are the main reasons for transplantation-related morbidity and mortality at early post-transplantation phases. Thus, much effort is currently being invested into the development of strategies to improve the efficacy of HSCT by increasing the numbers of transplanted cells (*i.e.* mobilization, *ex vivo* expansion). Here, we investigated whether inhibition of apoptosis could serve as a strategic alternative to increase the efficacy of stem cell transplantations. We focused on the role of BH3-only proteins, central regulators of the intrinsic apoptosis pathway, and identified Bim and Puma as rate limiting for cell death under conditions of cytokine deprivation *in vitro* (Fig 1B), consistent with findings previously made in different lymphocyte subsets (Erlacher et al, 2006; You et al, 2006). Focusing on the role of Bim in HSPC transplantation experiments *in vivo*, we observed superior reconstitution of *bim*^*−/−*^ HSPC in competitive transplantation experiments matching those overexpressing Bcl-2 (Fig 2). In contrast, loss of Bmf, that can act with Bim in a redundant manner in some forms of cell death (Hubner et al, 2010) and is strongly induced in HSPCs *in vitro* after factor deprivation, provided a more restricted advantage in this competitive setting (Fig 2). Importantly, the better performance of *bim*^*−/−*^ and *bmf*^*−/−*^ cells was not simply due to better survival and accumulation of peripheral leukocytes but was already evident at the HSPC level within the first days after transplantation (Fig 3C and D). Thus, HSPC apoptosis during early engraftment appears to be mediated by Bim and Bmf. Consistently, time to haematopoietic regeneration was clearly shortened in the absence of Bim or Bmf and significantly fewer *bim*^*−/−*^ HSPCs were required for successful reconstitution (Fig 4C). In light of these findings we propose that loss of Bim or Bmf leads to an extension of HSPC lifespan when these cells are deprived of their physiological microenvironment. Thus, their time available for homing to stem cell niches is extended and a higher proportion of HSPCs can repopulate the recipient's BM over time. Our *in vitro* work suggests that the increased resistance to cytokine deprivation may be causative for the superior engraftment of *bim*^*−/−*^ LSK cells. The importance of Bim for factor deprivation-mediated cell death has been extensively documented in different cell types (Pinon et al, 2008), yet we are the first to describe its role in HSPC death. In contrast, loss of Bmf failed to rescue HSPCs from factor deprivation-induced death *in vitro* (Fig 1B). Therefore, an alternative explanation for the *in vivo* reconstitution advantages of *bmf*^*−/−*^ HSPCs is required. Bmf has been implicated in apoptosis caused by loss of integrin signalling in certain cell types (Puthalakath et al, 2001) and hence may limit survival of HSPCs expelled from the niche. Loss of Bmf could thereby result in increased numbers of stem cells able to home to the BM. Accordingly, the noted upregulation of Bmf mRNA in human and murine HSPCs may be triggered by a combination of lack of growth factors and adhesion. Of note, our observations are in line with a recently published work showing that overexpression of miR-125b results in enhanced HSPC function and increased fitness during transplantations, and which suggested that the target genes responsible for these effects may be Bmf and/or KLF13 (Ooi et al, 2010). Yet no formal proof was provided that manipulation of Bmf levels would be causal for the effects seen in response to miR-125b overexpression. Our work now provides strong evidence that indeed loss of Bmf can increase HSPC fitness during transplantation and thereby nicely complements the work of Ooi et al (2010).

Based on the data available, it may not come as a surprise that 16 weeks after transplantation almost no wt leukocytes were present in wt:*bim*^*−/−*^ and wt:*bcl-2 tg* BM chimeras. However, the proportional as well as numerical regression of wt LSK cells over time was unexpected given their proposed cell death resistance and low turn over rate (Wilson et al, 2009). A possible explanation would be that Bcl-2 regulated cell death is triggered by various negative feedback signals coming from the replenished periphery and hitting the HSPC pool. Such feedback signals may lead to Bim-dependent apoptosis aimed to counteract excessive HSPC expansion during haematopoietic regeneration. Alternatively, as HSPCs seem to permanently egress from and resettle into stem cell niches, either in a division-dependent manner or under steady-state conditions (Bhattacharya et al, 2009), Bim-deficient or Bcl-2 overexpressing HSPCs may outcompete wt cells in this process due to their longer lifespan in the absence of niche-derived signals. In contrast to Bim, Bmf seems to be less critical in regulating long-term haematopoiesis as *bmf*^*−/−*^ donor cells were unable to completely displace wt cells within the time frame of the experiment, although in competitive assays *bmf*^*−/−*^ cells clearly performed better than their wt counterparts. Remarkably, the Ly5.2/Ly5.1 ratio in the HSPC pool remained stable in wt:*bmf*^*−/−*^ chimeric mice indicating an early but transient survival benefit during reconstitution (Fig 3D).

Despite promising results during early engraftment, the observed long-term displacement of wt cells by apoptosis-resistant counterparts could be hazardous: overshooting haematopoietic expansion may increase the risk of tumourigenesis and both *bim*^*−/−*^ and *vav-bcl-2 tg* mice show an increased incidence of tumours when paired with aberrant oncogene activation and are prone to develop signs of autoimmunity (reviewed by Strasser, 2005). However, in the absence of aberrant oncogene activation or deletion of additional cell death regulators, malignant transformation of Bim-deficient or Bcl-2 transgenic lymphocytes is a rare and late event (Egle et al, 2004; Erlacher et al, 2006; Strasser et al, 1990). Consistently, no leukaemias or lymphomas arose in recipient mice over an observation period of 8 months. Admittedly, this does not exclude the formal possibility that such pathologies may occur at later stages after transplantation and this will be subject of future studies.

At first sight it may seem surprising that *bim*^*−/−*^, *bmf*^*−/−*^ or other BH3-only protein-deficient mice do not show an accumulation of HSPCs in their BM, although they accumulate mature lymphocytes over time. However, one reason may be that apoptosis is a subordinate mechanism in regulating the stem cell pool in the absence of intense proliferation demand or other stress signals. Notably here, stem cell numbers are slightly increased in *vav-bcl-2 tg* as well as *H2K-bcl-2 tg* animals (Fig 1D and Domen et al, 2000; Ogilvy et al, 1999). This suggests that a combination of two or more BH3-only proteins is involved in the regulation of the HSPC pool under steady state conditions or, equally possible, loss of an individual BH3-only protein, such as Bim, does not cause an increase in freely available critical pro-survival molecules that match the levels achieved in transgenic Mcl-1 or Bcl-2 mice. As a consequence, redundantly acting BH3-only proteins, or compensatory upregulation may be able to maintain HSPC numbers under steady state conditions. In support of the latter, we noted highly elevated Bmf mRNA levels in the *bim*^*−/−*^ LSK cells (Supporting Information Fig 1B).

Importantly, our *in vitro* and *in vivo* experiments performed on human cord blood-derived CD34^+^ cells suggest that BIM and BMF are important for the regulation of HSPC homeostasis in both species (Figs 6 and 7 and Supporting Information Figs 6 and 7). The inferior effect of BIM RNAi on the survival of CD34^+^ progenitors in culture may ultimately be due to the incomplete knockdown achieved. Along similar lines the lack of *in vitro* cell death inhibition upon BMF depletion needs to be seen in relation to the comparatively mild effect of complete loss of Bmf on HSPC homeostasis in mice. The displacement of Ly5.1^+^ wt cells by Ly5.2^+^ *bim*^*−/−*^ or *bmf*^*−/−*^ cells observed in our *in vivo* murine transplantation model can nicely be translated to our xenograft model, where knockdown of BIM or BMF leads to a displacement of GFP-negative cells. Importantly, this already takes place in the immature CD34^+^ cell pool (Fig 7 and Supporting Information Figs 6 and 7).

Taken together, our data indicates that modulation of Bim or Bmf levels inhibits apoptosis in murine and human HSPCs and that the resulting extended life span is beneficial during HSCT, both during long-term engraftment and early phases of regeneration. We provide evidence that inhibition of the intrinsic apoptosis pathway could serve as a suitable therapeutic option to increase resistance of human HSPCs to factor deprivation and other types of stress caused during HSCT. However, since permanent apoptosis inhibition in lymphocytes and/or their progenitors can trigger lymphadenopathy, thereby increasing the residual risk of malignant transformation of these cells over time, such inhibition needs to be transient, when used therapeutically in order to satisfy biomedical and bioethical safety issues. Yet, with improved siRNA delivery technology and adenoviral vector systems that allow integration-independent transient expression of target genes at hand, it should be possible to overcome the limitations of our proof-of-principle approach. These issues are subject of ongoing investigations in our laboratory.

## MATERIALS AND METHODS

### Mice

All experiments were performed according to the guidelines of the Austrian and German ‘Tierversuchsgesetz’ and approved by the local committees (BMWF-66.011/0165-II/3b/2010; Austria; G09-40, RP Freiburg/Germany). Generation and genotyping of *vav-bcl-2 tg*, *bmf*^*−/−*^, *bim*^*−/−*^, *puma*^*−/−*^, *noxa*^*−/−*^ and *bad*^*−/−*^ mice has been described previously (Bouillet et al, 1999; Labi et al, 2008; Ogilvy et al, 1999; Ranger et al, 2003; Villunger et al, 2003). All mice were maintained on a C57BL/6 background.

### Cell isolation and culture

Murine lin^−^sca-1^+^c-kit^+^ cells (LSK) were isolated by magnetic bead-depletion of lin^+^ cells (Dynabeads) and subsequent sorting of viable (7-AAD^−^) sca-1^+^c-kit^+^ cells using a FACSvantage cell sorter (BD). lin^+^ cells were stained with the following antibodies: anti-Nk1.1 PK136, anti-Gr-1 RB6-8C5, anti-CD8 53-5.8, anti-CD4 GK1.5, anti-Mac1 M1/70, anti-B220 RA3-6B2, anti-Ter119 (Biolegend and eBioscience). LSK cells were cultured in RPMI1640 medium (PAA) supplemented with l-glutamine (Gibco), 2-mercaptoethanol, non-essential amino acids (Gibco), penicillin/streptomycin (Sigma), FCS (PAA) and cytokines (Preprotech). For Ly5.1/Ly5.2 staining of LSK CD150^+^ cells, magnetic beads enriched lin^−^ cells were enriched for c-kit^+^ cells by using anti-mouse c-kit magnetic particles (BD) for positive selection, and finally stained with antibodies against Sca1, CD150, Ly5.1 and Ly5.2.

Human umbilical cord blood was obtained immediately after caesarean birth. Informed consent was given by the parents and approval by the local ethics committee. After density gradient centrifugation, CD34^+^ cells were enriched by the MACS-technology. Purity of cells was evaluated by flow cytometry and generally >90%. Isolated cells were cultured in serum-free medium supplemented with ES-FBS (Invitrogen) and the indicated cytokines (Immunotools, Preprotech). Cells were cultured at a density of 10^5^–10^6^/ml. Alternatively, purified cells were frozen in serum/10% DMSO, stored in liquid nitrogen and used at later time points.

### Lentiviral vectors

Lentiviruses were generated by using pLeGOhU6-G, a third-generation lentivector, in which the murine U6 promoter had been exchanged for its human counterpart and which harbours an eGFP marker (Roelz et al, 2010). Oligonucleotides containing the 19-bp shRNAs (Supporting Information Table I), a nine-nucleotide loop and a pol-III-terminator sequence were cloned into pLeGOhU6-G. Additionally, pLeGO-iG vectors were used for generation of Bcl-2 and Bcl-x_L_ expressing lentiviruses. Lentiviruses were produced by HEK293T cells co-transfected with a vsv.g-envelope and a gag/pol plasmid. CD34^+^ cells were incubated for 48 h with viruses (2 × 24 h, MOI = 10). For *in vitro* experiments eGFP^+^ cells were FACS-sorted 24 h after 2nd infection. RNAi efficacy was determined by qRT-PCR and immunoblotting. Lentiviral work was performed under S2 safety conditions.

### Transplantation assays

Total BM or LSK cells were transplanted in 200 µl i.v. into lethally irradiated (10 Gy) Ly5.1^+^C57BL/6 mice 6–8 h post irradiation. For competitive reconstitution assays, 15,000 Ly5.1^+^ LSK cells and 15,000 Ly5.2^+^ LSK cells derived from different genotypes were mixed and transplanted into irradiated Ly5.1^+^ mice. Recipients were sacrificed at indicated time points and haematological organs were analysed in detail. Limiting dilution assays: 10,000 Ly5.2^+^ total BM cells derived from indicated genotypes were transplanted simultaneously with 200,000 Ly5.1^+^ competitor cells (total BM). Successful engraftment was defined by >1% Ly5.2^+^ myeloid and >1% Ly5.2^+^ lymphatic splenic cells 14 weeks later (Akala et al, 2008). Serial transplantations: for primary transplantations, total BM cells of indicated genotypes were transplanted into Ly5.1^+^ recipients. Secondary transplantations were performed by injecting 5000 Ly5.2^+^ LSK cells isolated 16 weeks after transplantation from primary recipients along with 200,000 Ly5.1^+^ total BM cells. Sixteen weeks after transplantation, Ly5.2^+^ LSK cells were isolated from secondary recipients, and tertiary transplantation was performed accordingly. Competitor Ly5.1^+^ total BM cells were always isolated from 9- to 11-week-old unchallenged mice.

### Homing assay

Fifteen hours after transplantation of 30,000 Ly5.2^+^ LSK into lethally irradiated mice, recipients were sacrificed and BM was flushed from femurs, tibias and iliac crests (‘central BM’). Additionally, endosteal cells were harvested by grinding the bones with a pestle and by subsequent digestion with collagenase. The two fractions were stained with anti-Ly5.1 and anti-Ly5.2 antibodies and analysed by flow cytometry.

### Xenograft assay

The protocol published by Traggiai et al (2004) has been used. Mice double deficient for rag2 and the IL-2 receptor γ-chain (rag2^*−/−*^γc^*−/−*^) and therefore lacking B, T and functional NK cells were irradiated with 2.5 Gy within their first week of life. 6–8 h later they were injected intrahepatically with the progeny of 10^5^ human CD34^+^ cells transduced twice with lentiviruses and cultured for 72 h. Cells were transplanted in a final volume of 25 µl. Alternatively, 7 weeks old mice were transplanted intravenously with the progeny of 10^5^ lentivirally transduced CD34^+^ cells (final volume 200 µl). Eight weeks after transplantation, animals were sacrificed for analysis. Single cell suspensions were obtained from BM, spleen and thymus. To obtain haematopoietic cells residing within the liver, tissue was digested with collagenase (1 mg/ml) and DNase (50 µg/ml) and density gradient centrifugation was performed subsequently.

**The paper explained**

PROBLEM:

At present, many haematological diseases can be cured only by transplantation of blood stem cells. This treatment is still associated with a significant risk of failure due to the loss of transplanted cells caused by poorly defined mechanisms. Clinical experience has shown that transplantation of higher stem cell numbers results in faster regeneration of the patient's bone marrow and reduces risk of graft failure. Therefore, much effort is put into the development of new strategies that increase donor stem cell numbers prior transplantation, for example by their *in vitro* expansion.

We hypothesized that a significant fraction of transferred stem cells undergoes cell death early during the procedure of transplantation due to the loss of survival signals usually provided by their natural environment, the stem cell niche. The aim of this study was to define molecules responsible for stem cell death during transplantation and to test whether cell death inhibition can increase transplantation efficacy.

RESULTS:

We identified two pro-apoptotic members of the Bcl-2 family, Bim and Bmf, as major factors limiting transplantation and long-term reconstitution efficacy of haematopoietic stem cells. Both proteins have previously been shown to play important roles in the homeostasis of mature lymphocytes. When Bim- or Bmf-deficient blood stem cells were transplanted simultaneously in a 1:1 ratio with cells from wild-type mice, these wild-type cells were readily displaced by the cell death-resistant stem cells as early as 10 days after transplantation. Furthermore, transplantation of Bim- or Bmf-deficient stem cells reduced the time required for successful regeneration of the recipient's blood system, and significantly lower numbers of Bim-deficient cells were required for reconstitution. Most importantly, we show that the function of Bim and Bmf is conserved between mice and humans, and that inhibition of either protein in human cord blood-derived stem cells provided a clear engraftment advantage in a xenotransplantation model.

IMPACT:

Our data indicate that apoptosis inhibition at the level of Bcl-2 family proteins results in an extended life span of mouse and human blood stem cells during transplantation and facilitates engraftment *in vivo*. Therefore, transient inhibition of cell death in human stem/progenitor cells could serve as a suitable therapeutic option to protect blood stem cells from transplantation stress.

### Clonogenicity assay

Colony forming assays were performed by plating hCD34^+^ on a semi-solid medium containing the indicated cytokines (MethoCult®). After incubating cells for 11 days at 37°C different types of colonies were quantified by light microscopy based on typical morphological features. After 14 days of culture cells were isolated and used for further experiments.

### Proliferation assay

LSK cells were isolated from donor mice (prior transplantation) or from recipient mice 4 weeks after non-competitive transplantation of 30,000 LSK cells. Cells were fixed with ice-cold ethanol (75%) and stained with FITC-labelled Ki-67 antibody and DAPI. To analyse cell cycle progression *in vitro*, freshly isolated LSK cells were stimulated with the cytokines TPO, SCF and Flt3L (100 ng/ml each) for 72 h before they were stained.

### Flow cytometric analysis

Single cell suspensions of haematopoietic organs were surface-stained with monoclonal antibodies conjugated with FITC, PE, APC or biotin. Antibodies for murine cell surface markers: D7, Sca-1; 2B8, c-kit; TC15-12F12.2, CD150; HM48-1, CD48; RA3-6B2, anti-B220; GK1.5, anti-CD4; YTS169, anti-CD8; RB6-8C5, anti-Gr-1; R2/60, anti-CD43; 5.1, anti-IgM; 11/26C, anti-IgD; MI/70, anti-Mac-1; Ter119; T24.31.2, anti-Thy-1; IM7, anti-CD44; H57-59, anti-TCRb; 154-2C11, anti-CD3; A20, anti-Ly5.1 and 104, anti-Ly5.2 (eBioscience or Biolegend). Lineage cocktail (eBioscience): RB6-8C5, Ter-119, RAB-6B2, 145-2C11, M1/70. Antibodies for human cell surface markers: HI30 and 30-F11, anti-CD45; AC136, anti-CD34. Biotinylated antibodies were detected using streptavidin-PE or -PECy7. Percentages of viable cells were determined by staining cells with 7-AAD and Annexin-V. Flow cytometric analysis was performed using a FACScalibur.

### Immunoblotting

Proteins were purified and size-fractioned by 12% SDS/PAGE under reducing conditions and transferred onto nitrocellulose membranes by electroblotting. Primary mouse or rat monoclonal antibodies were used (3C5, Bim; 9G10, BMF; 44, Bcl-xL; Bcl-2/100, Bcl-2; Alexis or BD). Secondary reagents were conjugated to peroxidase and signal was detected by enhanced chemiluminescence.

### RT-MLPA and qPCR

RT-MLPA: RNA from 100,000 to 200,000 cells was isolated by using Fast-Spin columns (ZymoResearch). RT-MLPA (MRC Holland, kit RM002, R011-B1 or R011-C1) was performed according to the manufacturer's instructions. Briefly, specific mRNAs were reversely transcribed into cDNA and bound by two oligonucleotides consequently ligated by a heat-stable ligase. Each probe gives rise to an amplification product of unique length separated by capillary sequencer (Genescan). Analysis was performed with GeneMapper (Applied Biosystems). The sum of all peak data was set to 100% to normalize for fluctuations between different samples, and single peaks were calculated relative to 100%.

qRT-PCR: RNA isolation (ZymoResearch) and cDNA synthesis (Biorad) were performed according to the manufacturer's instructions. qRT-PCR was performed using a Mastercycler Gradient (Eppendorf), the DyNAmo-Flash SYBR mastermix (Finnzymes) and primers indicated in Supporting Information Table I. Results were normalized to β-actin (mouse) or 18S expression (human) and evaluated using the −ΔΔ*C*_T_ relative quantification method.

### Statistical analysis

Statistical analysis was performed using the unpaired Student-*t*-test with Welch's correction, the Mann–Whitney-test or the Fisher exact test as indicated (Statview 4.1 software program). *p* values <0.05 were considered as statistically significant.

## Supplementary material

Detailed facts of importance to specialist readers are published as ”Supporting Information”. Such documents are peer-reviewed, but not copy-edited or typeset. They are made available as submitted by the authors.
