# Supplementary material for: Haematopoietic stem cell survival and transplantation efficacy is limited by the BH3-only proteins Bim and Bmf
Source: EMBO Mol Med. 2012 Nov 24;5(1):122–36. doi: 10.1002/emmm.201201235 (PMC3569658; doi:10.1002/emmm.201201235)
Supplement: Supplementary file 1 [file emmm0005-0122-SD1.pdf]

EMBO Molecular Medicine

Manuscript EMM-2012-01235

Haematopoietic Stem Cell Survival and Transplantation Efficacy is Limited by the BH3-only Proteins Bim and Bmf

Haematopoietic Stem Cell Survival and Transplantation Efficacy is Limited by the BH3-only Proteins Bim and Bmf

Verena Labi, Daniela Bertele, Claudia Woess, Denise Tischner, Florian J. Bock, Sven Schwemmers, Heike L. Pahl, Stephan Geley, Mirjam Kunze, Charlotte M. Niemeyer, Andreas Villunger and Miriam Erlacher

Corresponding author: Miriam Erlacher, University Hospital of Freiburg

Review timeline:

Submission date:	25 January 2012
Editorial Decision:	15 February 2012
Revision received:	13 August 2012
Editorial Decision:	04 September 2012
Revision received:	05 October 2012

Transaction Report:

(Note: With the exception of the correction of typographical or spelling errors that could be a source of ambiguity, letters and reports are not edited. The original formatting of letters and referee reports may not be reflected in this compilation.)

1st Editorial Decision 15 February 2012

Thank you for the submission of your manuscript to our editorial office. We have now received the enclosed reports.

As you will see, Referee #2 is rather positive about it. The other two reviewers, while considering the results potentially interesting, raise serious concerns regarding the conclusiveness of the data and medical relevance in this form and pinpoint several shortcomings that preclude a solid interpretation of the experimental data. The reviewers call for a considerable amount of additional work to resolve these issues in term of number of animals to study (to increase the statistic power of your analysis, as this was pointed out by all three reviewers) but also length of the analysis.

Regarding the latter, we particularly find the potential lymphoma development a serious enough issue that we believe the animals have to be monitored for a much longer time period to assess safety as recommended by Referee #3. In addition longer time analysis would also benefit the interpretation of long-term repopulation of HSC (Referees #2 and #3) and engraftment potential using adults Rag2-/-gc-/- rather than newborns as recommended by Referee #3.

Given the nature of these criticisms, the amount of work and time likely to be required to address them, and the fact that EMBO Molecular Medicine can only invite revision of papers that receive enthusiastic support from a majority of referees, I am afraid that we do not feel it would be productive to call for a revised version of your manuscript at this stage and therefore we cannot offer

to publish it.

However, because of the potential interest of the findings, we would have no objection to consider a new manuscript on the same topic if at some time in the near future you obtained data that would considerably strengthen the message of the study and address the referees concerns in full. To be completely clear, however, I would like to stress that if you were to send a new manuscript this would be treated as a new submission rather than a revision and would be reviewed afresh, in particular with respect to the literature and the novelty of your findings at the time of resubmission. If you decide to follow this route, please make sure you nevertheless upload a letter of response to the referees' comments.

At this stage of analysis, though, I am sorry to have to disappoint you. I nevertheless hope, that the referee comments will be helpful in your continued work in this area and I thank you for considering EMBO Molecular Medicine.

Yours sincerely,

Editor
EMBO Molecular Medicine

***** Reviewer's comments *****

Referee #1 (Comments on Novelty/Model System):

See major comment below

Referee #1 (Other Remarks):

In their manuscript, Labi claim that suppressing two BH3-only proteins, Bim and Bmf, will protect Hematopoietic Stem Cell (HSC) from apoptosis and so increase transplantation efficiency. The first part concerning mouse cells is rather convincing. They show that cytokine deprivation increases the expression of several BH3-only proteins and decreases Bcl2 in purified LSK. Moreover, competitive reconstitution and limiting dilution assay show an advantage of bim -/- or, at lesser extent, Bmf-/- cells, that recapitulates bcl2 overexpression phenotype as early as 10 days after transplantation. The results with human Cord Blood by lentivirally transduced sh RNA transplanted into newborn rag2-/-gammac-/- mice are less convincing with a very high variability in the data combined to a wrong statistical analysis (especially at the view of the CD34 staining of the "representative experience"). Although this work is important for deciphering apoptosis pathways in HSC survival, I don't think it has an important medical impact for now, especially, as the authors said in their last sentence, considering the high risk of lymphoma-genesis and autoimmunity due to the role of these proteins in B cell survival and autoreactive T cell selection.

Major comments

This represents a huge amount of work that deserves at my view a more rigorous analyze. First, the number of independent data point is sometimes low (less than 3) and so the authors definitely need to use another statistic test than the student t test that required both a normal distribution and an equal variance. Use of non-parametric test such as Wilcoxon would have been more appropriate (especially that in most of the case the data are paired).
The authors should limit data on competitive reconstitution to HSC and progenitors, since they already published data on B and T cells in their 1998 J exp med paper.

Minor comments

Why Puma was not studied after the 1st paragraph?
Table 1 contains too much data to be clear despite lacking statistics

Referee #2:

General Comments

The manuscript by Labi et. al., from the laboratories of Erlacher and Labi presents new, interesting, and potentially translatable findings. The impact of the studies is enhanced by a clearly-written and thoughtout manuscript with data from mice using knock-out strategies, and from human cord blood using knock-down strategy. The engraftment data is convincing. However, there are a number of comments below that the investigators need to address.

Specific Comments - Major

1. Page 9, second paragraph: Secondary transplants should be done if the primary mice are still available, as this will add to information on the long-term repopulating HSC population, and to possible effects on self-renewal of the transplanted cells.

2. It is important to know if any of the engrafting differences are due to changes in the homing capacities of the transplanted cells. Do short-term homing assays (e.g. 24 hours) for the knock-out cells and analyze engraftment for different populations (LSK, CD150+LSK, etc.).

Other Comments

1. Add statistics for Supplemental Figures (e.g. SFig 1A).

2. Table I is too complicated. Try to simplify. You can remove all percentage data, and focus on absolute cell numbers. Provide statistical analysis for the numbers.

3. Page 8, 18 lines from top: It is extremely rare that CD34+ cells are used for HSCT grafts, so remove this statement.

4. Page 10, lines 6 & 7 from bottom: This statement: "...first to describe a key role..." is not clear. Clarify.

5. The legend to Suppl. Fig. 3 cites this as Fig. 2. Fix.

Referee #3 (Comments on Novelty/Model System):

The data are interesting, however I have major concerns about whether most of the phenotype is due to a pre-leukaemia rather than true HSC self-renewal. This is especially important given that both Bmf and Bim knockouts have been shown to more readily develop lymphoma. The human data is interesting, but again a bit too preliminary and more data required.

Referee #3 (Other Remarks):

In this manuscript the authors explore the role of pro-apoptotic, the "BH-3 only", proteins in hematopoietic stem and progenitor cell (HSPC) homeostasis. The authors demonstrate that in vitro cytokine deprivation resulted in mRNA expression level changes for both anti and pro apoptotic proteins in HPSC. Bim-/- or Bcl-2 over expressing HSPC showed both early and improved in vivo hematopoietic reconstitition potential along with decreased apoptosis in vitro. Furthermore, Bim-/- or Bcl-2 over expressing bone marrow showed superior haematopoietic reconstitution potential by limiting dilution assay. Lastly, authors demonstrate superior in vitro survival and in vivo reconstitution potential of human cord blood derived HSPC with RNAi based kockdown for Bim or BMF gene using xenograft mice transplantation model. I have some comments regarding this manuscript as follows:

Major concerns:

1) Given that loss of Bim or Bmf have been shown to increase risk of lymphoma from developing, further long-term (up to and including 12 months post-transplant) analysis of the recipient mice is important to determine whether these mice developed (which may in turn have influenced results as pre-leukaemic cells can inaccurately represent HSC self-renewal). The congenic mouse transplants were only investigated up to 16 weeks post-transplant, and the rag2-/-gc-/- recipients only to 8 weeks post-transplant. Much longer analysis should be performed.

2) The authors show that Bim-/- LKS+ cells have enhanced long-term haematopoietic reconstitution potential compared to wild type LKS+ cells which is a result of increased survival of HSPC. However, they do not provide any information about the cell cycle status of Bim-/- HSCs. Is the enhanced long-term reconstition potential of HSC at least partly due to altered cell cycling or proliferative potential of Bim-/- HSPC?

3) In Fig 4C, the numbers of mice used for the limiting dilution assay were insufficient for correct statistical analysis. Furthermore, as indicated in the methods section, analysis of these mice was performed at 14 weeks post-transplant, which is too early to monitor long-term repopulation. Experiments should be performed with larger numbers of mice to enable better analysis of data using Poisson statistics.

4) Control CB HSPC that have not been transduced with vectors are not included in their data for Figs 6 (exception of 6B) and 7. It is important, especially for the rag2-/-gc-/- transplant, to determine what the non-transduced CB HSPC repopulating activity was in these mice.

5) The authors have used newborn Rag2-/-gc-/- mice in their in vivo xenograft transplantation experiment. Usage of adult Rag2-/-gc-/- mice would have mimicked the adult BM microenvironment and also provided a more stringent model for assessment of haematopoietic engraftment potential.

6) In the Rag2-/-gc-/- transplant analysis only CD45+ and CD34+ cells are analysed. A more complete analysis of lineage cells is required.

Minor concerns:

1) The authors have mentioned that cytokine deprivation of LKS+ cells resulted in no change in the mRNA expression level of bik gene but has not included the data for the same in Figure 1A.

2) It appears the authors largely analysed B cells only using IgM (see suppl Fig 2). Given that in their previous publication (Labi et al J. Exp Med 205:641-655, 2008) they have shown that both Bmf-/- and Bim-/- mice have increased numbers of immature B cells in their BM and spleen it is important to analyse all mice using both B220 and IgM. The data for B and T lymphocytes in the bone marrow of the transplanted mice should be included in this figure. Furthermore, the profiles for Mac-1/Gr-1 look very strange- did they exclude erythroid cells by gating out FSC/SSC in the analysis? In the bone marrow there is usually a distinct Gr-1+Mac-1+ population that is very bright (mature granulocytes), and a dimmer Gr-1+ population that is Mac-1+ (immature granulocytes). It appears that in their recipients of bim-/- cells the Gr-1 bright population is absent, but this needs to be better analysed to be sure. If this holds true this is of concern re. potential leukaemia development. The profiles of WT and bim-/- definitely look very different.

3) Representative FACS profiles of the different lineages within the donor-derived populations in the peripheral blood of the transplanted mice would be important to include in supplemental Figure for all strains of mice.

1st Revision - authors' response 13 August 2012

******* EMM-2012-01235: Reviewer's comments *******
Point-to-point reply:

Referee #1

In their manuscript, Labi claim that suppressing two BH3-only proteins, Bim and Bmf, will protect Hematopoietic Stem Cell (HSC) from apoptosis and so increase transplantation efficiency. The first part concerning mouse cells is rather convincing. They show that cytokine deprivation increases the expression of several BH3-only proteins and decreases Bcl2 in purified LSK. Moreover, competitive reconstitution and limiting dilution assay show an advantage of bim -/- or, at lesser extent, Bmf-/- cells, that recapitulates bcl2 overexpression phenotype as early as 10 days after transplantation.
The results with human Cord Blood by lentivirally transduced sh RNA transplanted into newborn rag2-/-gammac-/- mice are less convincing with a very high variability in the data combined to a wrong statistical analysis (especially at the view of the CD34 staining of the "representative experience").

Reply: We appreciate the positive evaluation of our mouse work that set the basis for highly relevant but more challenging experiments using human cord-blood derived $CD34^+$ progenitor cells. As noted also by other researchers in the field, xenograft mouse models tend to show a rather high variability in engraftment (see: Brehm et al., Clin.Immunol, 2010; Strowig et al, PNAS, 2011; Rozemuller, Exp.Hematol., 2004). To overcome this obstacle and to address the concern related to the statistical analysis, we significantly increased the number of xenotransplant experiments using up to 14 recipient mice/genotype and, after consulting with our biostatistician, applied the non-parametric Mann-Whitney test to explore statistically significant differences.
In addition, according to the Referee's suggestion we have re-evaluated our FACS data and Fig. 6 now shows updated versions of primary dot plots of CD34 stainings derived from our xenograft assays 8 weeks after transplantation. Furthermore, Suppl. Fig. 7 shows CD34+ stainings obtained from reconstitution experiments using adult $rag2^{-/-}gc^{-/-}$ recipients.

Although this work is important for deciphering apoptosis pathways in HSC survival, I don't think it has an important medical impact for now, especially, as the authors said in their last sentence, considering the high risk of lymphomagenesis and autoimmunity due to the role of these proteins in B cell survival and autoreactive T cell selection.

Reply: We agree with Referee #1 that our work has no "immediate therapeutic impact" in terms that this strategy would be applicable immediately in the clinics. Yet, with all due respect, we do not consider this a strong enough argument that should preclude publication of our findings that may pave the way for future clinical application. We clearly demonstrate that apoptosis limits the efficacy of stem cell transplantations early on during transplantation mainly via the activation of Bim and Bmf and our work provides the basis for further follow-up studies aiming for the development of novel or application of existing short-term acting apoptosis inhibitory strategies that function at the level of the "Bcl-2 rheostat".
In relation to the valid concern that deregulation of the "Bcl2 rheostat" in lymphocytes can foster pathology such as cancer or autoimmunity, we have performed a series of experiments that demonstrate that even under conditions of continuous deregulation of these molecules for up to 8 month (not intended in therapeutic settings, of course), in the context of

reconstitution of Ly5.1 recipients, no evidence of pathology was observed. Given that this represents about 1/3 of the natural life-span of a laboratory mouse, we believe that this provides sufficient evidence that *transient* inhibition of these molecules in a therapeutic setting can be considered reasonably safe. Suffice to say that this clearly requires additional prolonged follow-up in future studies, but hopefully may satisfy this immediate concern, shared also by the editor and Referee #3.

Major comments
This represents a huge amount of work that deserves at my view a more rigorous analyze. First, the number of independent data point is sometimes low (less than 3) and so the authors definitely need to use another statistic test than the student t test that required both a normal distribution and an equal variance. Use of non-parametric test such as Wilcoxon would have been more appropriate (especially that in most of the case the data are paired).

Reply: We appreciate that this referee recognizes the huge amount of work involved in the experiments described in our manuscript. This analysis has now been extended even further into different cell types using neonatal and now also adult xenograft models. As far as possible in the given time frame for revision, the number of independent data points has been increased, and the non-parametric Mann-Whitney Test has been applied for all xenograft experiments where a normal distribution cannot be expected. The results of these experiments are now presented in the new version of Fig. 7 and Suppl.Fig. 6+7 of the revised version of the manuscript.

The authors should limit data on competitive reconstitution to HSC and progenitors, since they already published data on B and T cells in their 1998 J exp med paper.

Reply: This referee rightly noted that we published first data on the reconstitution potential of Bmf-deficient bone marrow in our 2008 JEM paper (Labi et al). However, as our current project was set out to correlate the production of mature cells with the status of immature stem and progenitor cells in direct comparison with other genotypes not investigated in the mentioned study, we still deem it appropriate to include data on mature T and B cells. We also believe that the use of different pools of cells for reconstitution, i.e. LSK (this study) vs. total bone-marrow (JEM 2008) actually requires showing these results, although parts only confirm our own published findings.

Minor comments
Why Puma was not studied after the 1st paragraph?

Reply: In 2010 we published a work on the role of Puma in haematopoietic stem and progenitor cell survival after radiation damage (Labi et a, Genes Dev.) where we also found evidence for possible novel functions of this BH3-only protein during stem cell regeneration. These findings are subject of a separate line of investigations that go beyond the scope of this manuscript but are still too preliminary to share with the scientific community.

Table 1 contains too much data to be clear despite lacking statistics

Reply: In order to simplify Table 1 we reduced the data shown to the ratio of Ly5.2+/Ly5.1+ cells found in recipient animals 16 weeks after reconstitution and included appropriate

statistical analysis (unpaired t-test).The absolute Ly5.1 and Ly5.2 cell numbers of all organs analyzed (including statistics) can now be found in Suppl.Table III.

Referee #2

General Comments
The manuscript by Labi et. al., from the laboratories of Erlacher and Labi presents new, interesting, and potentially translatable findings. The impact of the studies is enhanced by a clearly-written and thoughtout manuscript with data from mice using knock-out strategies, and from human cord blood using knock-down strategy. The engraftment data is convincing.

Reply: We would like to thank Referee #2 for his/her very positive evaluation of our work.

However, there are a number of comments below that the investigators need to address.
Specific Comments - Major
1. Page 9, second paragraph: Secondary transplants should be done if the primary mice are still available, as this will add to information on the long-term repopulating HSC population, and to possible effects on self-renewal of the transplanted cells.

Reply: Since we were fortunate enough that secondary transplantation experiments were already ongoing when we first submitted our work, we can now include data on secondary and even tertiary transplantation experiments. The results of these experiments are presented in the new version of Suppl.Fig.4. We can show that despite resistance against apoptosis, LSK cells lacking *bim*$^{-/-}$, *bmf*$^{-/-}$ or that overexpress transgenic *bcl-2* exhaust at similar rates and that the overall stemness of these cells is not affected by loss of apoptotic capacity, a finding we consider rather remarkably. Furthermore, in contrast to published literature (i.e.: Kamminga, Stem Cells, 2005; Nakamura, PLoS One, 2012) we were not able to transplant cells 4-5 times. We believe that this discrepancy is most likely due to the method of serial transplantation: in contrast to other studies we used sorted Ly5.2+ LSK cells and Ly5.1+ total bone marrow cells of young mice as competitors which had not been transplanted serially. Thus, the Ly5.1+ bone marrow used must be considered highly competitive.
Due to the limited time available for revision we were unable to repeat this type of experiment for a third time and to further increase the n-numbers (now ranging between and n=3-6 for secondary transplantation), but we believe that we provide sufficient evidence to proof our point.

2. It is important to know if any of the engrafting differences are due to changes in the homing capacities of the transplanted cells. Do short-term homing assays (e.g. 24 hours) for the knock-out cells and analyze engraftment for different populations (LSK, CD150+LSK, etc.).

Reply: This is a valid concern and important question. Homing of wt and Bim-deficient LSK cells in the central bone marrow and in the endosteum was analyzed 15 hours after transplantation by adopting the protocol published by Williams and Nilsson (2009). We did not observe significant differences between genotypes. The results are now presented in the new version of Suppl.Fig.3 and on page 7 of the results section.

Other Comments
1. Add statistics for Supplemental Figures (e.g. SFig 1A).

Reply: The qRT-PCR analysis was only performed twice (in duplicate), we therefore did not perform statistical analysis, but decided to present the mean values of this analysis as an additional line of evidence confirming our findings made using RT-MLPA analysis. We did not generate additional biological replicates to extend our qRT-PCR analysis. We hope that this referee will find our way of data presentation satisfactory. If preferred, we can show the results of the individual experiments separately.

2. Table I is too complicated. Try to simplify. You can remove all percentage data, and focus on absolute cell numbers. Provide statistical analysis for the numbers.

Reply: In order to simplify Table 1, also requested by Referee #1, we decided to show the ratio Ly5.2+/Ly5.1+ cells and included appropriate statistics (unpaired Students t-test). For convenience, the absolute cell numbers of all organs analyzed can now be found in Suppl.Table III.

3. Page 8, 18 lines from top: It is extremely rare that CD34+ cells are used for HSCT grafts, so remove this statement.

Reply: The sentence has been changed to: “CD34+ cells… used as a source of HSCT grafts in certain transplantation regimens such as haplo-identical transplantations” (page 8).

4. Page 10, lines 6 & 7 from bottom: This statement: "...first to describe a key role..." is not clear. Clarify.

Reply: To make our point clearer we changed the sentence to: “The importance of Bim for factor deprivation-mediated cell death has been extensively documented in different cell types, yet we are the first to describe its role in HSPC death”.

5. The legend to Suppl. Fig. 3 cites this as Fig. 2. Fix.

Reply: The legend has been changed.

Referee #3:
In this manuscript the authors explore the role of pro-apoptotic, the "BH-3 only", proteins in hematopoietic stem and progenitor cell (HSPC) homeostasis. The authors demonstrate that in vitro cytokine deprivation resulted in mRNA expression level changes for both anti and pro apoptotic proteins in HPSC. Bim-/- or Bcl-2 over expressing HSPC showed both early and improved in vivo hematopoietic reconstitition potential along with decreased apoptosis in vitro. Furthermore, Bim-/- or Bcl-2 over expressing bone marrow showed superior haematopoietic reconstitution potential by limiting dilution assay. Lastly, authors demonstrate superior in vitro survival and in vivo reconstitution potential of human cord blood derived HSPC with RNAi based kockdown for Bim or BMF gene using xenograft mice transplantation model. I have some comments regarding this manuscript as follows:
The data are interesting, however I have major concerns about whether most of the

phenotype is due to a pre-leukaemia rather than true HSC self-renewal. This is especially important given that both Bmf and Bim knockouts have been shown to more readily develop lymphoma.

Reply: We are glad to see that Referee #3 also considers our work as interesting and appreciate his/her concern related to a possible increase in tumorigenesis, caused by lack of Bmf or Bim (also noted by Referee #1). However, we believe that this concern is overrated as we (and others) have shown that the incidence of spontaneously arising tumors in unchallenged Bim-deficient mice is truly minor (Egle 2004; Erlacher 2006) and basically negligible in Bmf deficient mice (Labi 2008, Baumgartner 2012). All donor mice were used at the age of 8-12 weeks and showed no signs of disease and normal bone-marrow subset composition, with the exception of the mild increase in mature lymphocyte numbers observed in the absence of Bim or when Bcl-2 is overexpressed (Bouillet 1999; Erlacher 2006). Also in our recipient mice, observed up to 8 months after transplantation, or in our serial transplantation assays (3x16 weeks of follow up) we did not observe a single case of lymphoma or leukemia. In addition, we breed Bim- and Bmf-deficient as well as transgenic mice in our facility since 2003 and never observed malignant disease in any of the mentioned genotypes before the age of about one year (VL, ME & AV, unpublished observations). Thus, we are confident that no pre-leukemic cells were transplanted in our experiments.

The human data is interesting, but again a bit too preliminary and more data required.

Reply: As suggested by this referee human data were extended to an adult xenograft model and to the analysis of differentiated B and myeloid cells. T cells were omitted from our presentation as in our analysis almost no T cells developed in recipient mice, in line with previously published results (Traggiai et al, Science, 2004). Furthermore, our analysis of newborn mice, reconstituted with CD34+ cells was extended to increased n-numbers as much as possible in the time-frame given for revision.

Referee #3 (Other Remarks):
Major concerns:
1) Given that loss of Bim or Bmf have been shown to increase risk of lymphoma from developing, further long-term (up to and including 12 months post-transplant) analysis of the recipient mice is important to determine whether these mice developed (which may in turn have influenced results as pre-leukaemic cells can inaccurately represent HSC self-renewal). The congenic mouse transplants were only investigated up to 16 weeks post-transplant, and the rag2-/-gc-/- recipients only to 8 weeks post-transplant. Much longer analysis should be performed.

Reply: As indicated above, we were fortunate enough that experiments addressing this question were started at the time of submission of our original manuscript. Therefore, we can refer to data obtained from monitoring recipient mice that were reconstituted with bone marrow derived from wt, Bim- or Bmf-deficient mice and now observed for 8 months after transplantation (n=10-14 per genotype). During this period, no signs of lymphoma, leukemia or autoimmunity developed in the recipient mice. Due to the time-frame given for revision, a longer follow up was not possible

Moreover, we performed serial transplantation assays, now shown in Suppl.Fig.4. Again, no malignancies or autoimmune diseases were observed in the recipient mice, independently of the donor genotype (3x16 weeks follow up).
Admittedly, in our xenograft models we mainly focused on early engraftment, although xenograft models can be used to analyze human engraftment up to 20 weeks (Notta/Dick, Science, 2011). We analyzed our cohorts at the time of 8 weeks post transplantation, as we believe that the average life span of *rag2*$^{-/-}$*gc*$^{-/-}$ recipient mice is simply too short and the number of human cells formed in these hosts to low to reliably detect spontaneous transformation of human cord-blood cells that show Bim knock-down or overexpress Bcl2. Nonetheless, we agree with this referee that we can not fully judge or exclude a residual risk inherent to our experimental approach, but believe that the robust assessment of such risk requires substantially larger cohorts of mice subjected to the maximal possible observation period that in our point of view is simply beyond the scope of this study and certainly exceeds the time-frame granted for revison.
Importantly, we want to emphasize again that the main aim of our study was not to demonstrate safety of ***permanent*** apoptosis inhibition but to show effectiveness of apoptosis inhibition throughout reconstitution and, most importantly, early during engraftment. Our data may hopefully lead to the development of more suitable ***short-acting*** apoptosis inhibitory regimens for the treatment of HSPCs that function at the level of the "Bcl-2 rheostat".

2) The authors show that Bim-/- LKS cells have enhanced long-term haematopoietic reconstitution potential compared to wild type LKS cells which is a result of increased survival of HSPC. However, they do not provide any information about the cell cycle status of Bim-/- HSCs. Is the enhanced long-term reconstition potential of HSC at least partly due to altered cell cycling or proliferative potential of Bim-/- HSPC?

Reply: This concern is well taken. In order to address this issue, we analyzed the cell cycle status by a combined Ki-67/DAPI stain. LSK cells were stained prior transplantation; LSK cells isolated from recipients 4 weeks after transplantation were subjected to the same procedure. No differences were observed between wt and *bim*$^{-/-}$ donor cells. We also assessed the capacity of wt and Bim-deficient LSK cells to progress through cell cycle after stimulation with TPO, SCF and Flt3L *in vitro*, but again failed to observe differences between the two groups. We therefore can exclude that increased proliferation of *bim*$^{-/-}$ contributes to the enhanced haematopoietic reconstitution potential. The results are shown in the new version of Suppl. figure 3 of the revised manuscript.

3) In Fig 4C, the numbers of mice used for the limiting dilution assay were insufficient for correct statistical analysis. Furthermore, as indicated in the methods section, analysis of these mice was performed at 14 weeks post-transplant, which is too early to monitor long-term repopulation. Experiments should be performed with larger numbers of mice to enable better analysis of data using Poisson statistics.

Rely: We agree with this Referee that the number of mice were insufficient to perform reliable Poisson statistics. However, we now increased the number of mice transplanted with 10,000 Ly5.2^{+} BM cells, where we had identified the most significant difference between wt and *bim*$^{-/-}$ donors in our initial experiments. We now show data representing the fact that 0/6 or 4/5 mice engrafted successfully, as defined by more than 1% myeloid and more than 1% lymphatic Ly5.2^{+} cells in the spleen at 14 weeks after reconstitution, when wt or *bim*$^{-/-}$ BM

donors where used, respectively. We believe that this finding provides sufficient evidence that Bim-deficient stem cells show superior reconstitution potential over wt cells, at least under the conditions tested. By using the Fisher exact test to determine the association between Ly5.2$^+$ engraftment and Bim deficiency, we obtained a highly significant p-value of p=0.015. Description of the experimental setup and data presentation has been updated accordingly in the revised version of the manuscript on page 8 and in Fig.4. Statistical analysis was supervised by an experienced biostatistician (Dr. Donna Neuberg, Dana Farber Cancer Institute, Boston) and deemed appropriate.
Concerning the time point of the analysis we want to point out that published literature indicates that short term (ST)-HSC can give rise to all lineages for 8-12 weeks, but at later time points mature cells arise exclusively from LT-HSC as described by Christensen and Weissman, 2001. For our study, we relied on the protocol published by Akala/Clarke (Nature 2008) where long-term multi-lineage reconstitution of recipient mice was analyzed as early as 14 weeks after transplantation.

4) Control CB HSPC that have not been transduced with vectors are not included in their data for Figs 6 (exception of 6B) and 7. It is important, especially for the rag2-/-gc-/- transplant, to determine what the non-transduced CB HSPC repopulating activity was in these mice.

Reply: Please see below data generated from mice that were reconstituted with non-transduced cord blood HSPC. These cells seem to perform better than cells that were transduced with control vectors (compare with Fig.7, right panel here), a finding in line with published literature that documents that viral transduction *per se* usually results in a decreased engraftment capacity of HSPC (Liu/Broxmeyer, Exp.Hematol., 2008). These data have not been included into Fig.7 for the sake of simplicity. However, all relevant controls (Luciferase-shRNA and empty vectors) have been included in our figures.

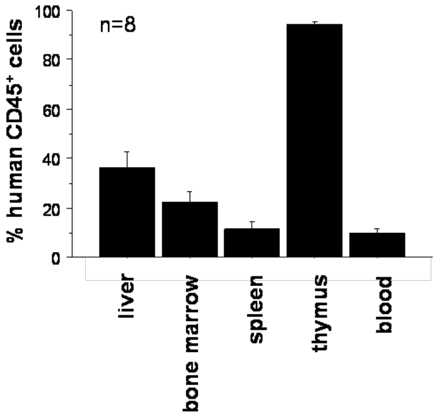

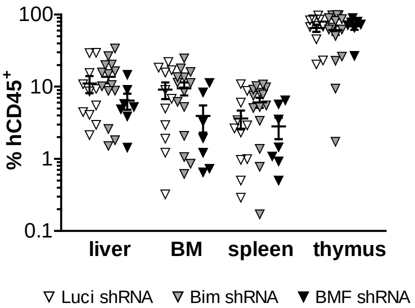

5) The authors have used newborn Rag2-/-gc-/- mice in their in vivo xenograft transplantation experiment. Usage of adult Rag2-/-gc-/- mice would have mimicked the adult BM microenvironment and also provided a more stringent model for assessment of haematopoietic engraftment potential.

Reply: We realized that this is a severe concern. In order to address this issue, we performed intravenous injection of human HSPC cells into adult *rag2$^{-/-}$gc$^{-/-}$* recipient animals that clearly

represents a superior, albeit less established model, for the assessment of engraftment potential of human HSPCs as it seems much closer to a possible clinical setting. The results of this analysis are presented in the new version of Suppl. Fig. 7 and in accordance with our transplantation experiments performed in newborn animals. Splenic engraftment was very low in adult recipients and was therefore not included in our analysis.
Generally, most authors use intraosseous transplantation in adult recipient mice in order to achieve a higher reconstitution. However, we decided to transplant intravenously to mimic haematopoietic engraftment best.

6) In the Rag2-/-gc-/- transplant analysis only CD45+ and CD34+ cells are analysed. A more complete analysis of lineage cells is required.

Reply: We now provide a more detailed data analysis that includes also the percentage of B and myeloid cells in the new version of Suppl.Fig.6+7 referring to newborn (Suppl.Fig.6) and adult recipients (Suppl.Fig.7), respectively. Representative dot plots of our FACS analysis are now also shown.

Minor concerns:
1) The authors have mentioned that cytokine deprivation of LKS cells resulted in no change in the mRNA expression level of bik gene but has not included the data for the same in Figure 1A.

Reply: We apologize for the misleading description of our results. In fact, bik mRNA is not detected in LSK cells by RT-MLPA analysis, neither in the presence nor in the absence of cytokines. This has been corrected.

2) It appears the authors largely analysed B cells only using IgM (see suppl Fig 2). Given that in their previous publication (Labi et al J. Exp Med 205:641-655, 2008) they have shown that both Bmf-/- and Bim-/- mice have increased numbers of immature B cells in their BM and spleen it is important to analyse all mice using both B220 and IgM. The data for B and T lymphocytes in the bone marrow of the transplanted mice should be included in this figure.

Reply: As shown in Figure 2 and Table 1, B220+IgM+ B cells have been analysed. Table 1 and Suppl. Table III contain additional information on BM preB cells and splenic B cell subsets, such as T1 and T2 cells. Generally, all subsets analyzed showed similar Ly5.2/1 ratios. Since the two other referees strongly suggested reducing data volume on mature cells and simplification of our figures we rather refrain from including additional data in our primary figures.

Furthermore, the profiles for Mac-1/Gr-1 look very strange- did they exclude erythroid cells by gating out FSC/SSC in the analysis? In the bone marrow there is usually a distinct Gr-1+Mac-1+ population that is very bright (mature granulocytes), and a dimmer Gr-1+ population that is Mac-1+ (immature granulocytes). It appears that in their recipients of bim-/- cells the Gr-1 bright population is absent, but this needs to be better analysed to be sure. If this holds true this is of concern re. potential leukaemia development. The profiles of WT and bim-/- definitely look very different.

Reply: We thank Referee #3 for his accuracy. After careful re-evaluation of myeloid subsets we are sure that Mac1/Gr1 expression is similar in wt:wt and wt:*bim*$^{-/-}$ chimeras and updated the relevant Suppl.Fig.2 accordingly, showing a more representative example.

3) Representative FACS profiles of the different lineages within the donor-derived populations in the peripheral blood of the transplanted mice would be important to include in supplemental Figure for all strains of mice.

Reply: Since Referee #1 and #2 suggested reducing data on mature cells we decided not to include additional figure panels.

2nd Editorial Decision 04 September 2012

Thank you for the submission of your revised manuscript "Haematopoietic Stem Cell Survival and Transplantation Efficacy is Limited by the BH3-only Proteins Bim and Bmf" to EMBO Molecular Medicine.

We have now received the enclosed reports from the referees that were asked to re-assess it. As you will see, the reviewers acknowledge that the manuscript was significantly improved during revision. However, while Reviewer #2 indicates that that the manuscript is suitable for publication, Reviewer #1 still raises an issue that should be convincingly addressed.

This Reviewer highlights again that the statistical analysis of the data should be improved. Please either use the non-parametric tests mentioned or justify the use of a test, which assumes normal distribution.

On a more editorial note, please address the following points:

- The description of all reported data that includes statistical testing must state the name of the statistical test used to generate error bars and P values, the number (n) of independent experiments underlying each data point (not replicate measures of one sample), and the actual P value for each test (not merely 'significant' or 'P < 0.05').

- Please improve the resolution of Fig 5C, which is currently too low for publication.

- We noted that you included figures in your point-by-point response. Do you agree that they will be published in the Review Process File?

Revised manuscripts should be submitted within one month of a request for revision; they will otherwise be treated as new submissions, unless arranged otherwise with the editor.

I look forward to seeing a revised form of your manuscript as soon as possible.

Yours sincerely,

Editor
EMBO Molecular Medicine

***** Reviewer's comments *****

Referee #1 (Comments on Novelty/Model System):

Labi et al clearly improved the second part of their manuscript by increasing the number of analyzed mice and adding the serial transplantation experiment. They clearly demonstrated a role of Bim in HSC survival during transplantation and so design this molecule as a target for improving efficacy of stem cell transplantation. The role of Bmf was less convincing especially according to the problems still present with the statistical analysis.

Referee #1 (Other Remarks):

Major Remarks

There is still a major problem with the statistical analysis. Although they rightfully used Mann-Whitney and Fisher tests in the last figures, there is no information about the test used in figure 1-6 (and supp fig 1-5). And since the given p values are the same than in the previous version of the

manuscript, one could only assume that they used student t test which was the only specified test of this first version. As I already said it in my first review, the authors cannot used this test due to the low number of independent observations, so they should do again the statistics using Mann-Whitney or Wilcoxon in case of paired data (such as in figure 1A).

Minor remark

In page 9, it's written that BMF induction was prevented by both SCF and Flt3L which I assume meant that they prevent BMF induction together, however, in fig 5B, they are together only in the presence of TPO and IL6 so the authors cannot rule out a role of these 2 other cytokines.

Referee #2 (Other Remarks):

None.

2nd Revision - authors' response 05 October 2012

As Reviewer 1 suggested we improved statistical analysis of our data. For all data sets we now used the non-parametric Mann-Whitney-Test. To this end, new experiments have been performed for Figure 1A to increase n-numbers ($n>3$ is required for significant p-values if the Mann-Whitney-Test is used). We now include data from $n=5$-6 from 4 independent experiments.

The overall conclusions drawn from these experiments stand unaltered.

An exception is Figure 1B where we used the Student's t-test with Welch correction which adjusts for inequality of variance by modifying the degrees of freedom. Thereby, this test can be used for comparison between mice of different genotypes where variability is unequal and sample size is small.

For all our analysis we have consulted with Dr. Donna Neuberg, a biostatistician at the Dana-Farber Cancer Institute with experience in animal studies.

We also changed the sentence on page 9 (BMF induction) according to the Referee's suggestion.

Furthermore, we adjusted figures and figure legends (description of statistical test, n of independent experiments, actual p-values), as requested by you. Fig. 5C has been exchanged to improve resolution.

Additionally, we agree that all our communication (including the figures in the point-by-point response) can be published in the Review Process File.

We hope that our efforts now satisfy the concerns raised by Referee1 and convince you that our work now meets all the high quality criteria to allow publication in EMM.
