# Supplementary material for: Haematopoietic stem cell survival and transplantation efficacy is limited by the BH3-only proteins Bim and Bmf
Source: EMBO Mol Med. 2012 Nov 24;5(1):122–36. doi: 10.1002/emmm.201201235 (PMC3569658; doi:10.1002/emmm.201201235)
Supplement: Supplementary file 2 [file emmm0005-0122-SD2.pdf]

Supplementary information

Haematopoietic Stem Cell Survival and Transplantation Efficacy is Limited by the BH3-only Proteins Bim and Bmf

Verena Labi*, Daniela Bertele*, Claudia Woess, Denise Tischner, Florian J. Bock, Sven Schwemmers, Heike L. Pahl, Stephan Geley, Mirjam Kunze, Charlotte M. Niemeyer, Andreas Villunger and Miriam Erlacher

Suppl. Table I	shRNA-sequences cloned into pLeGOhU6-G vectors and primers used for qRT-PCR
Suppl. Table II	MLPA data for murine LSK and human CD34$^+$ cells subjected to cytokine withdrawal *in vitro*
Suppl. Table III	Cell composition in wt:*bmf*$^{-/-}$, wt:*bim*$^{-/-}$ and wt:*bcl-2 tg* BM chimeras 16 weeks after competitive transplantation
Suppl.Fig. 1	qRT-PCR data for Bcl-2 family members in murine LSK cells subjected to cytokine withdrawal (A/B)<br>Analysis of %Ly5.2$^+$ LSK cells 5 days after competitive transplantation
Suppl.Fig. 2	Representative dot plots and gates used for data analysis
Suppl.Fig. 3	Homing and proliferation experiments performed with wt and Bim-deficient LSK cells
Suppl.Fig. 4	Serial transplantations of wt, *bim*$^{-/-}$, *bmf*$^{-/-}$ and *bcl-2 tg* LSK cells
Suppl.Fig. 5	Cytokine-deprivation induced apoptosis of human CD34$^+$ cells (A),<br>qRT-PCR for BIM, PUMA and BMF in human CD34$^+$ cells (B),<br>Western blot showing efficient downregulation of either BIM or BMF as well as overexpression of BCL-XL or BCL-2 in human CD34$^+$ cells (C)
Suppl.Fig. 6	Analysis of CD19$^+$ B and CD33$^+$ myeloid cells arising in *rag2*$^{-/-}$ *γc*$^{-/-}$ mice reconstituted intrahepatically with human cord blood derived HSPCs
Suppl.Fig. 7	Analysis of *rag2*$^{-/-}$ *γc*$^{-/-}$ mice transplanted intravenously with human CD34$^+$ cells

shRNA sequences:

BIM	TGATGTAAGTTCTGAGTGTG
BMF	TCAGCCGACTTCAGCTCTT
Luciferase	CGCTGAGTACTTCGAAATGTC

qRT-PCR primers (5‘-3‘):

actin (mu.)	sense	ACTGGGACGACATGGAGAAG
	antisense	GGGGTGTTGAAGGTCTCAAA
bad (mu)	sense	GGACTTATCAGCCGAAGCAG
	antisense	GCTCAAACTCTGGGATCTGG
bcl-2 (mu)	sense	CTGGCATCTTCTCCTTCCAG
	antisense	GACGGTAGCGACGAGAGAAG
bcl-xL (mu)	sense	TTCGGGATGGAGTAAACTGG
	antisense	TGGATCCAAGGCTCTAGGTG
bim (mu)	sense	GAGATACGGATTGCACAGGA
	antisense	TCAGCCTCGCGGTAATCATT
bmf (mu)	sense	CCCATAAGCCAGGAAGACAA
	antisense	AGGGAGAGGAAGCCTGTAGC
mcl-1 (mu)	sense	TAACAAACTGGGGCAGGATT
	antisense	GTCCCGTTTCGTCCTTACAA
noxa (mu)	sense	CCCACTCCTGGGAAAGTACA
	antisense	AATCCCTTCAGCCCTTGATT
puma (mu)	sense	CAAGAAGAGCAGCATCGACA
	antisense	TAGTTGGGCTCCATTTCTGG
18S (hu)	sense	TCAAGAACGAAAGTCGGAGG
	antisense	GGACATCTAAGGGCATCACA
BimEL (hu)	sense	TGACACAGACAGGAGCCCAGC
	antisense	CGCCGCAACTCTTGGGCGAT
BMF (hu)	sense	GTGCTCGTCACGCTGGACCC
	antisense	GGTCACCGGCTCCCCATCCT

Suppl.Table I:
shRNA-sequences cloned into pLeGOhU6-G vectors and primers used for qRT-PCR are indicated (mu: murine; hu: human).

murine
LSK cells

	+ Cytokines		- Cytokines		
	Mean	SEM	Mean	SEM	p value
Bcl-w	1	0.13	0.76	0.32	0.72
FLIP	1	0.17	0.89	0.24	0.86
Bcl-g	1	0.47	0.30	0.30	0.36
Bcl-rambo	1	0.12	0.95	0.04	0.20
MOAP-1	1	0.21	0.53	0.15	0.06
APAF1	1	0.20	1.22	0.23	0.36
AIF	1	0.24	0.62	0.09	0.20
XIAP	1	0.35	1.81	0.26	0.07
Survivin	1	0.28	0.65	0.07	0.27
BIRC1A	1	0.31	2.01	0.36	0.10
CIAP1	1	0.34	2.35	0.68	0.07
CIAP2	1	0.20	1.28	0.06	0.07
BIRC6	1	0.10	1.25	0.21	0.72
OMI	1	0.24	0.70	0.24	0.14
B2M	1	0.08	1.42	0.34	0.27
P21	1	0.21	0.90	0.31	0.86
Smac/Diablo	1	0.45	0.43	0.43	0.47
PAK2	1	0.45	0.38	0.24	0.41

human
CD34+ cells

	+ Cytokines		- Cytokines		
	Mean	SEM	Mean	SEM	p value
DR6	1	0.08	1.04	0.09	0.57
P21	1	0.12	0.54	0.10	0.02
Omi	1	0.10	1.26	0.06	0.11
Bcl-w	1	0.09	1.12	0.19	0.85
FLIP	1	0.12	2.18	0.19	0.002
Bcl-rambo	1	0.03	1.11	0.06	0.18
Birc1	1	0.22	3.94	0.80	0.002
Birc2	1	0.18	1.66	0.08	0.03
Birc3	1	0.12	1.29	0.12	0.22
XIAP	1	0.04	1.20	0.08	0.08
Survivin	1	0.09	0.27	0.02	0.002
Birc6	1	0.27	0.93	0.06	0.22
Serpin-B	1	0.07	0.95	0.04	0.95
PARN	1	0.24	0.71	0.05	0.18
AIF	1	0.06	0.80	0.07	0.14
GUSB	1	0.09	1.16	0.04	0.06
Bnip-3L	1	0.19	4.13	0.35	0.002
Apaf1	1	0.11	1.49	0.15	0.02
Perforin	1	0.54	0.86	0.58	0.75
Bnip-3	1	0.25	1.29	0.14	0.11
Smac/Diablo	1	0.07	0.84	0.06	0.18
Fas	1	0.13	1.64	0.32	0.14

Suppl.Table II:
Upper table: Murine wt LSK cells were isolated and cultured for 14h in the presence or absence of SCF, TPO and Flt3L (100 ng/ml each). mRNA levels of the indicated genes were determined by MLPA (n=5-6, 4 independent experiments). Lower table: Fresh human $CD34^{+}$ cells were cultured for 14h in the presence or absence of SCF, IL6, Flt3L (100 ng/ml each) and TPO (10 ng/ml). mRNA levels of the indicated genes were determined by MLPA (n=7, 5 independent experiments, Mann-Whithney-Test) but not confirmed by qRT-PCR.

		wt:wt	wt:*bmf*$^{-/-}$		wt:*bim*$^{-/-}$		wt:*bcl-2 tg*	
**BONE MARROW**								
Cell no. (2 femura)	x 10^7	7.93 ±0.56	8.37 ± 1.63	p=0.88	7.12 ± 1.20	0.46	8.88 ± 1.33	0.90
Ly5.1$^+$ LSK	x 10^4	8.29 ±2.30	4.55 ± 0.28	0.10	2.09 ± 0.83	0.03	1.81 ± 0.51	0.01
Ly5.2$^+$ LSK	x 10^4	9.13 ±2.46	11.50 ± 0.84	0.18	17.40 ± 3.97	0.14	14.36 ± 3.11	0.09
Ly5.1$^+$ LSK CD150$^+$	x 10^3	15.66 ±6.76	11.97 ± 5.35	0.88	3.10 ± 1.04	0.01	2.28 ± 0.52	0.01
Ly5.2$^+$ LSK CD150$^+$	x 10^3	16.69 ±7.22	34.88 ± 11.27	0.10	20.04 ± 6.43	0.62	17.21 ± 4.83	0.62
Ly5.1$^+$ preB	x 10^6	1.60 ±0.30	0.95 ± 0.34	0.18	0.25 ± 0.08	0.01	0.17 ± 0.02	0.01
Ly5.2$^+$ preB	x 10^6	2.41 ±0.47	2.48 ± 0.83	0.65	5.53 ± 2.28	0.62	10.49 ± 3.41	0.01
Ly5.1$^+$ monocytes	x 10^6	6.34 ±1.39	3.84 ± 1.22	0.30	1.07 ± 0.34	0.01	1.55 ± 0.17	0.01
Ly5.2$^+$ monocytes	x 10^6	8.14 ±2.31	14.09 ± 2.60	0.10	15.60 ± 5.37	0.09	12.92 ± 1.36	0.14
Ly5.1$^+$ granulocytes	x 10^6	11.20 ±1.93	5.42 ± 1.26	0.05	1.11 ± 0.18	0.01	3.05 ± 0.54	0.01
Ly5.2$^+$ granulocytes	x 10^6	15.47 ±1.93	21.67 ± 6.14	0.46	15.19 ± 4.08	0.81	19.43 ± 2.82	0.22
**THYMUS**								
Thymocyte no.	x 10^7	9.5 ±1.65	12.04 ± 2.25	0.76	4.62 ± 1.62	0.09	13.91 ± 4.60	0.46
Ly5.1$^+$ DN	x 10^6	1.18 ±0.21	0.98 ± 0.27	0.65	0.022 ± 0.006	0.01	0.060 ± 0.023	0.01
Ly5.2$^+$ DN	x 10^6	1.77 ±0.35	2.69 ± 0.14	0.03	3.84 ± 1.59	0.22	17.72 ± 8.48	0.01
Ly5.1$^+$ DP	x 10^6	17.96 ±3.47	18.16 ± 5.80	0.88	0.11 ± 0.05	0.01	0.58 ± 0.27	0.01
Ly5.2$^+$ DP	x 10^6	51.57 ±12.37	73.52 ± 24.57	0.65	25.08 ± 8.93	0.14	70.36 ± 23.14	0.46
Ly5.1$^+$ CD4$^+$	x 10^6	3.81 ±0.99	3.19 ± 0.61	0.65	0.077 ± 0.031	0.01	0.20 ± 0.07	0.01
Ly5.2$^+$ CD4$^+$	x 10^6	9.98 ±2.26	11.76 ± 4.15	0.88	12.38 ± 4.06	0.33	31.91 ± 8.87	0.14
Ly5.1$^+$ CD8$^+$	x 10^6	1.34 ±0.36	0.79 ± 0.15	0.46	0.013 ± 0.005	0.01	0.066 ± 0.022	0.01
Ly5.2$^+$ CD8$^+$	x 10^6	2.40 ±0.54	2.69 ± 0.58	0.88	4.04 ± 1.50	0.22	16.17 ± 6.58	0.01
**SPLEEN**								
Cell no.	x 10^7	11.11 ±1.51	17.64 ± 1.46	0.03	20.69 ± 4.74	0.09	22.01 ± 4.90	0.14
Ly5.1$^+$ CD4$^+$	x 10^7	0.63 ±0.17	0.83 ± 0.12	0.46	0.38 ± 0.27	0.46	0.14 ± 0.03	0.09
Ly5.2$^+$ CD4$^+$	x 10^7	1.03 ±0.15	2.08 ± 0.29	0.03	3.12 ± 0.64	0.01	4.09 ± 0.61	0.01
Ly5.1$^+$ CD8$^+$	x 10^7	0.41 ±0.10	0.67 ± 0.12	0.30	0.28 ± 0.19	0.22	0.12 ± 0.04	0.03
Ly5.2$^+$ CD8$^+$	x 10^7	0.70 ±0.16	1.04 ± 0.09	0.10	1.72 ± 0.44	0.09	2.28 ± 0.56	0.09
Ly5.1$^+$ B220$^+$IgM$^+$	x 10^6	22.32 ±4.76	21.55 ± 1.51	0.65	3.87 ± 0.55	0.01	3.15 ± 0.84	0.01
Ly5.2$^+$ B220$^+$IgM$^+$	x 10^6	33.07 ±7.16	63.01 ± 6.49	0.03	66.50 ± 12.56	0.09	47.08 ± 9.79	0.14
Ly5.1$^+$ T1	x 10^6	1.91 ±0.51	1.74 ± 0.27	0.65	0.20 ± 0.04	0.01	0.18 ± 0.09	0.01
Ly5.2$^+$ T1	x 10^6	3.71 ±0.84	8.70 ± 2.42	0.10	11.28 ± 4.27	0.09	11.94 ± 4.22	0.09
Ly5.1$^+$ T2	x 10^6	3.86 ±0.79	4.05 ± 2.15	0.65	0.67 ± 0.24	0.01	0.47 ± 0.18	0.01
Ly5.2$^+$ T2	x 10^6	7.47 ±1.48	11.53 ± 0.18	0.03	15.17 ± 6.02	0.33	9.67 ± 2.27	0.46
Ly5.1$^+$ monocytes	x 10^6	2.11 ±0.56	1.90 ± 0.52	0.65	0.64 ± 0.25	0.05	0.82 ± 0.33	0.14
Ly5.2$^+$ monocytes	x 10^6	2.00 ±0.55	4.71 ± 0.99	0.05	7.95 ± 2.91	0.03	6.48 ± 2.21	0.14
Ly5.1$^+$ granulocytes	x 10^6	0.93 ±0.24	0.33 ± 0.06	0.05	0.13 ± 0.05	0.01	0.27 ± 0.10	0.03
Ly5.2$^+$ granulocytes	x 10^6	0.73 ±0.18	0.99 ± 0.06	0.46	2.16 ± 0.56	0.01	1.73 ± 0.58	0.22
Ly5.1$^+$Ter119$^+$	x 10^6	4.25 ±1.23	3.84 ± 1.81	0.88	0.25 ± 0.05	0.01	0.51 ± 0.24	0.03
Ly5.2$^+$Ter119$^+$	x 10^6	4.35 ±1.65	9.74 ± 6.28	0.45	12.20 ± 3.58	0.14	17.62 ± 7.02	0.09
**BLOOD**								
WBC number	/µl	23320 ±1480	16433 ± 5182	0.18	17900 ± 5121	0.54	26175 ± 2550	0.46
Ly5.1$^+$ CD4$^+$	/µl	1003 ±131	428 ± 131	0.03	99 ± 31	0.01	181 ± 40	0.01
Ly5.2$^+$ CD4$^+$	/µl	1622 ±254	1150 ± 277	0.30	1736 ± 605	0.99	3286 ± 1277	0.33
Ly5.1$^+$ CD8$^+$	/µl	1266 ±184	592 ± 135	0.03	152 ± 47	0.01	360 ± 85	0.01
Ly5.2$^+$ CD8$^+$	/µl	1274 ±148	824 ± 207	0.10	2270 ± 686	0.22	3052 ± 300	0.01
Ly5.1$^+$ B220$^+$ IgM$^+$	/µl	510 ±98	180 ± 40	0.05	39 ± 18	0.01	90 ± 45	0.01
Ly5.2$^+$ B220$^+$ IgM$^+$	/µl	824 ±190	1077 ± 367	0.30	577 ± 283	0.46	634 ± 174	0.46
Ly5.1$^+$ monocytes	/µl	2452 ±748	1156 ± 539	0.30	253 ± 128	0.05	759 ± 310	0.14
Ly5.2$^+$ monocytes	/µl	1994 ±579	2220 ± 1040	0.45	3110 ± 1630	0.62	4030 ± 2070	0.46
Ly5.1$^+$ granulocytes	/µl	1019 ±467	156 ± 57	0.30	248 ± 39	0.62	319 ± 137	0.46
Ly5.2$^+$ granulocytes	/µl	1087 ±534	246 ± 84	0.30	1410 ± 259	0.62	1088 ± 467	0.62

Suppl.Table III:

Cell composition in wt:*bmf*$^{-/-}$, wt:*bim*$^{-/-}$ and wt:*bcl-2 tg* BM chimeras

16 weeks after competitive transplantation recipient mice were sacrificed, and all indicated haematopoietic organs were analyzed in detail by flow cytometry using cell surface marker-specific antibodies. Absolut cell numbers are depicted. Values represent means of n=4-6 animals per genotype from 4 independent experiments (+/- SEM). All p-values are indicated (Mann-Whitney-Test).

Labi_et_al_Suppl_Fig1

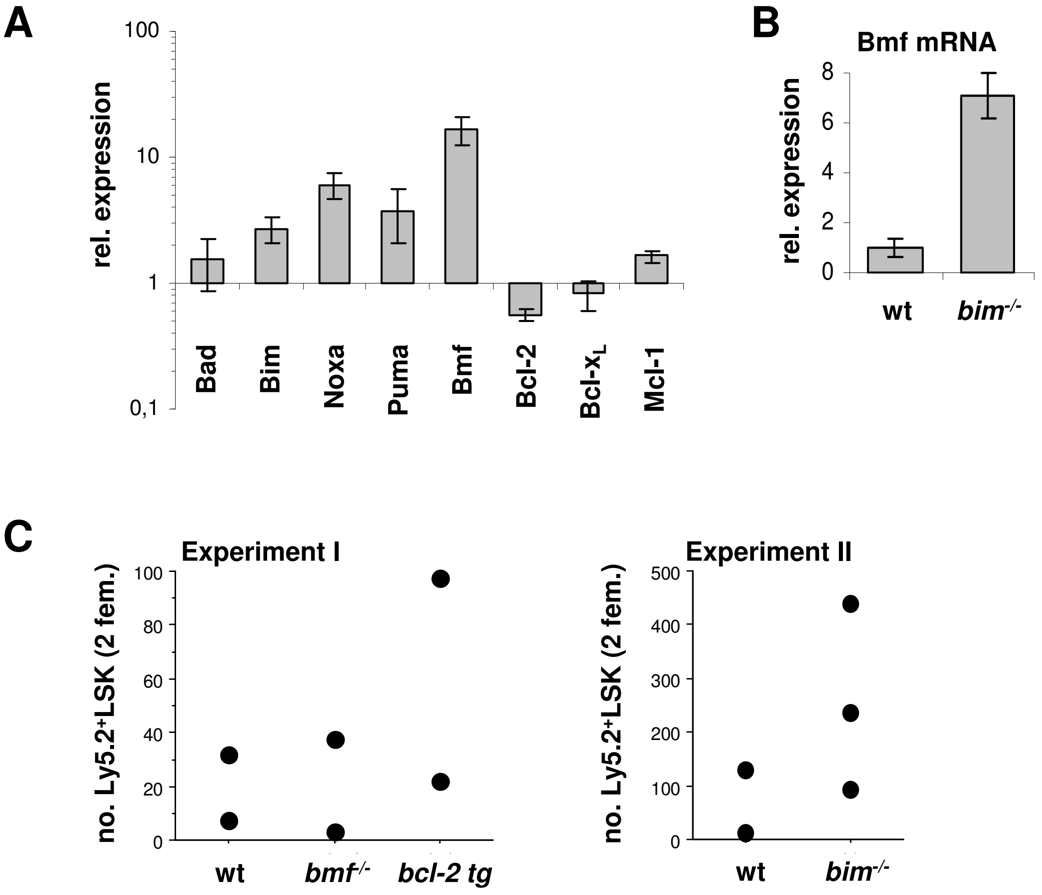

Suppl.Figure 1:

(A) Wild type LSK cells were isolated and cultured for 14h in the presence or absence of SCF, TPO and Flt3L (100 ng/ml each). mRNA levels of the indicated Bcl-2 family members were determined by qRT-PCR. Bars represent means of two independent experiments ±SD.

(B) Bmf expression was analyzed in freshly isolated wt and *bim*$^{-/-}$ LSK cells by MLPA, results were confirmed by qPCR (not shown). n=3 per genotype, no significant differences were obtained with the Mann-Whitney-Test.

(C) 5 days after competitive reconstitution, more *bim*$^{-/-}$ and *bcl-2 tg* than wt Ly5.2$^+$ LSK cells could be collected from the recipient's bone marrows. Experiments were highly variable (2 independent experiments shown).

Labi_et_al_Suppl_Fig2

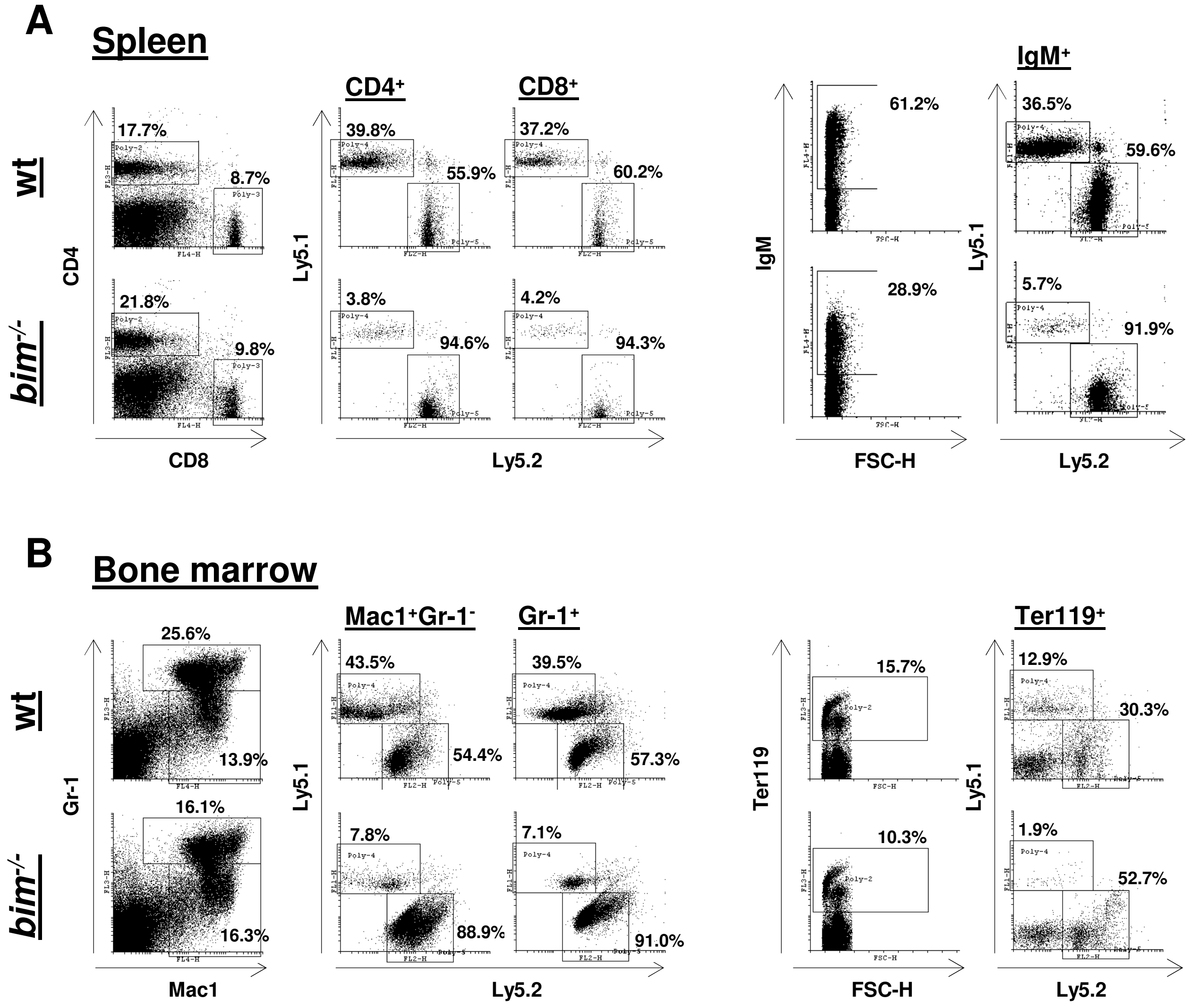

Suppl.Figure 2:
Representative dot plots and the gates used for analysis of T, B and myelo-erythroid cell populations are shown for splenic (A) or BM cells (B) isolated from wt and *bim*$^{-/-}$ mice.

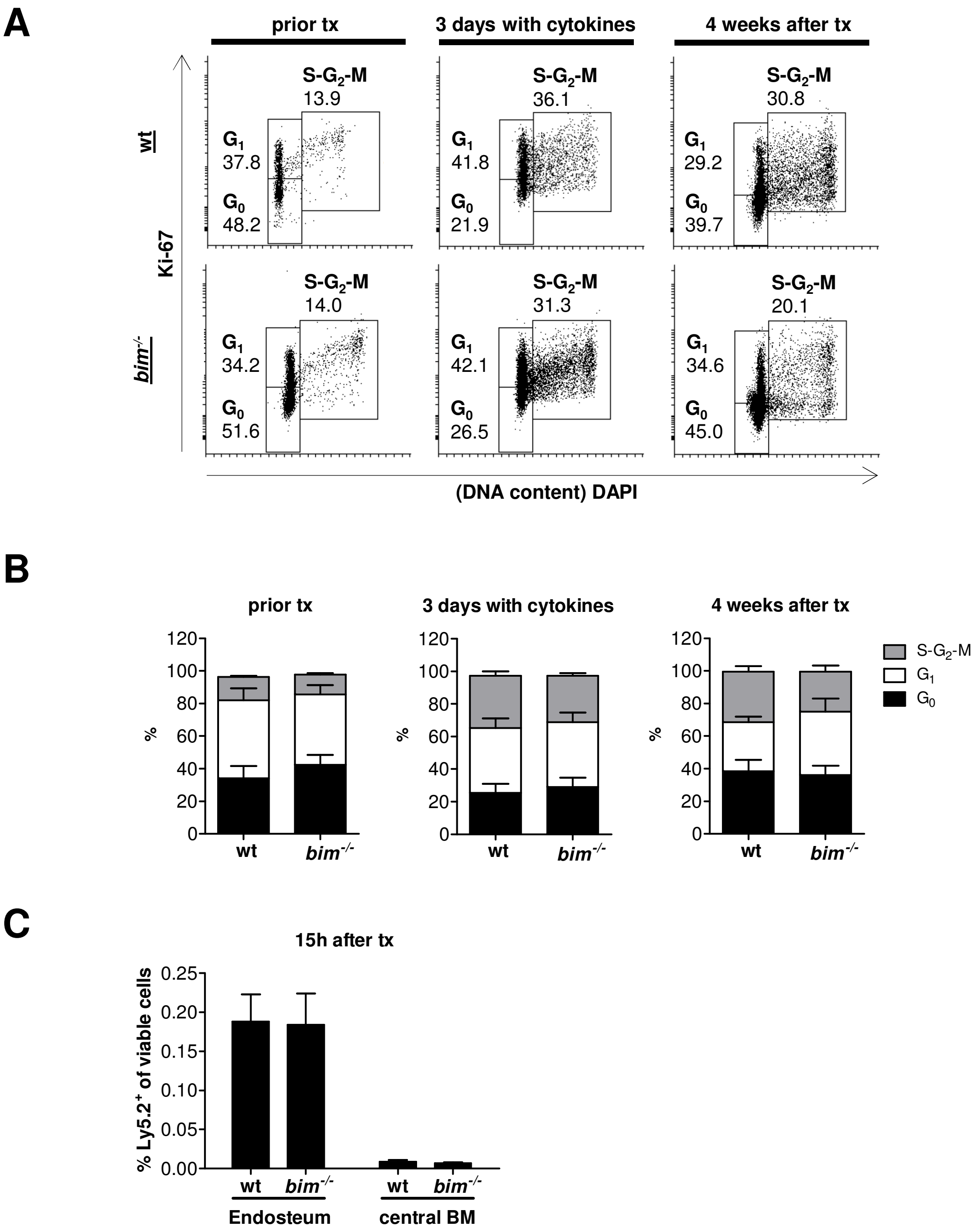

Suppl.Figure 3:
(A/B) Wt and *bim*$^{-/-}$ LSK cells were isolated from donor mice before transplantation (left, n=3) or from recipient mice 4 weeks after transplantation (right, n=3-4) and stained for Ki-67and DAPI. To test *in vitro* proliferation, LSK cells were cultured for 72 hours in the presence of TPO, SCF and Flt3L (middle, n=4). Representative dot plots are shown (A). Bars represent means +/- SEM from 2 independent experiments, no significant differences were obtained (Mann-Whitney-Test) (B).
(C) To test for homing potential, recipient mice were sacrificed 15 hours after transplantation with LSK cells derived from the indicated genotypes. BM fractions were isolated as described in the method section. % Ly5.2^{+} cells were determined by flow cytometry. Bars represent means +/- SEM; n=6-8 from 2 independent experiments. No signficant differences between genotypes were observed (Mann-Whitney-Test).

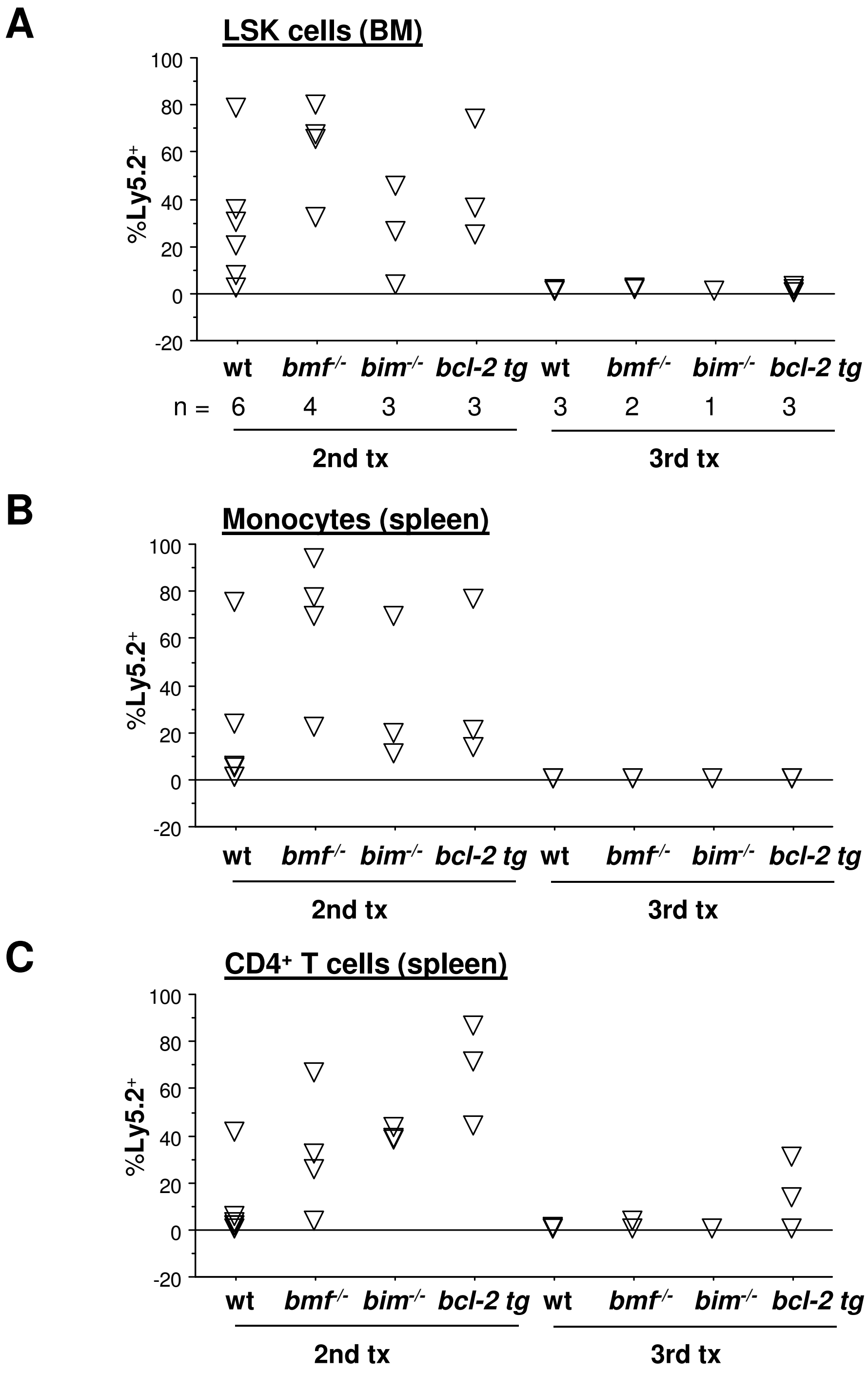

Suppl.Figure 4:
Serial transplantations were performed as indicated in the method section at 16 weeks intervals (5,000 Ly5.2$^+$ LSK cells + 200,000 Ly5.1$^+$ total BM cells). Since competing BM cells were derived from young, unchallenged Ly5.1 mice they were highly competitive. Whereas Ly5.2$^+$ LSK cells successfully reconstituted all secondary recipients, no Ly5.2$^+$ engraftment could be measured following tertiary transplantation, independently of the donor genotype. Graphs show % Ly5.2$^+$ cells within the LSK cell pool (A), within the splenic monocyte population (B) and within the splenic CD4$^+$ T cell population (C). Two independent experiments have been performed, n-numbers are indicated in the upper panel . Significant difference has been observed between wt and *bcl-2 tg* CD4$^+$ T cells in the spleen during 2nd transplantation (p=0.02, Mann-Whitney-Test).

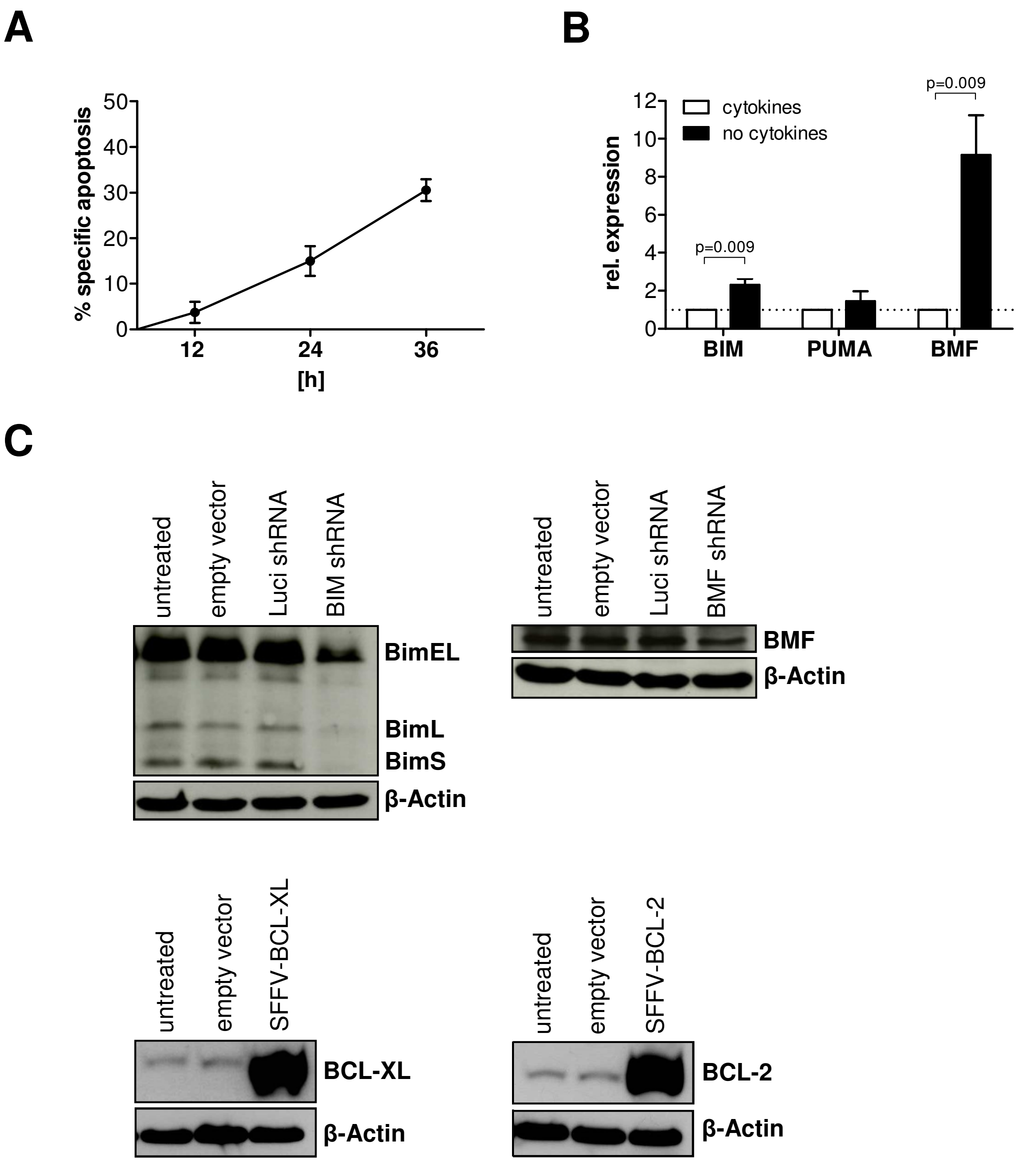

Suppl.Figure 5:

(A) Fresh cord blood derived CD34+ cells were cultured in the presence or absence of SCF, IL6, Flt3L (100 ng/ml each) and TPO (10 ng/ml). Cytokine-deprivation induced apoptosis was determined by combined staining with AnnexinV and 7-AAD and subsequent flow cytometry at the indicated time points. (B) Upregulation of BIM and BMF mRNA induced by cytokine deprivation observed in MLPA (Fig.5) were confirmed by qRT-PCR. Bars show means of n=3-5 from 3 independent experiments ±SEM, significant p-values are indicated (Mann-Whitney-Test) (C) Western blots performed with lentivirally transduced cord blood CD34+ cells showed an efficient downregulation of either BIM or BMF as well as overexpression of BCL-XL or BCL-2.

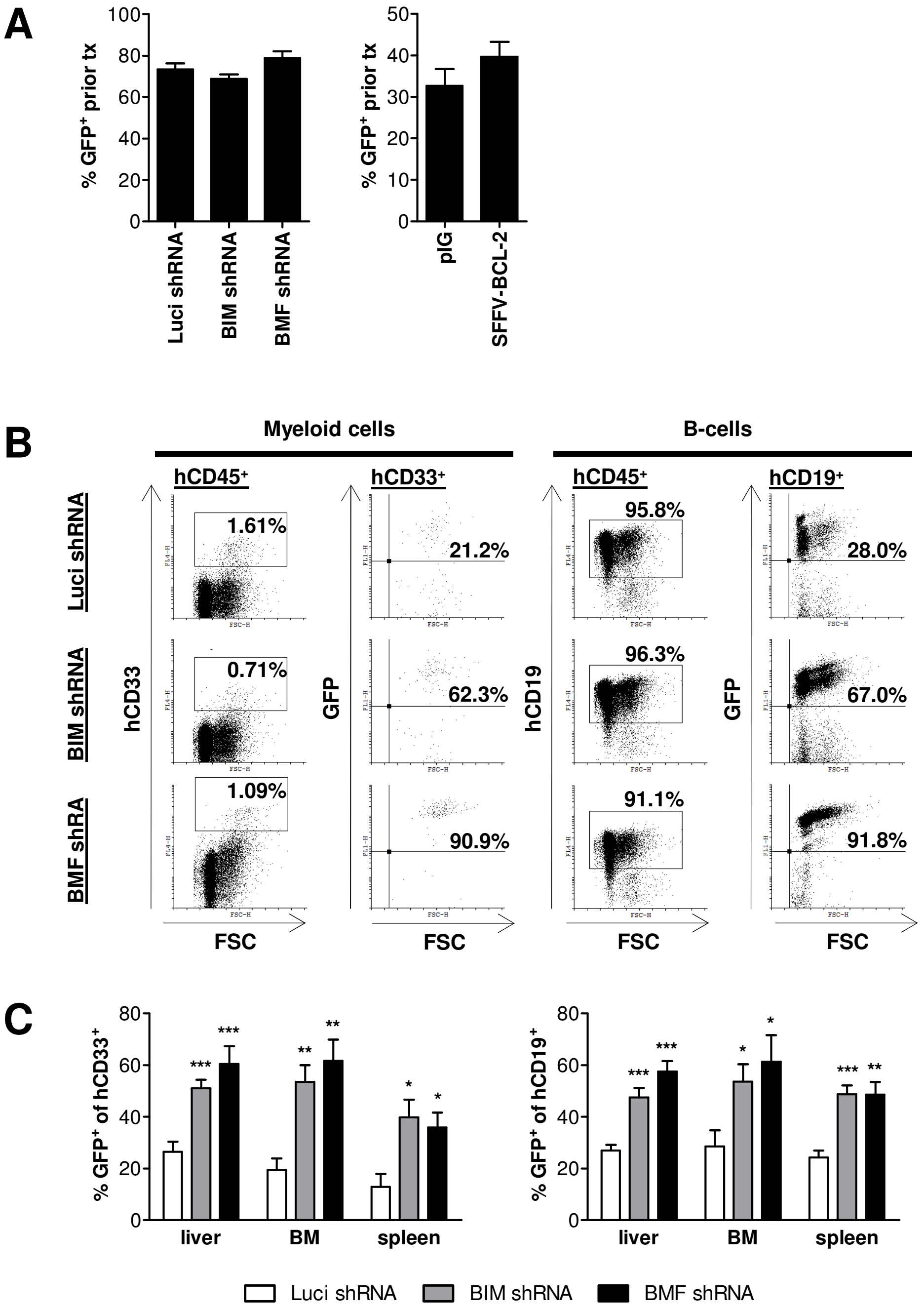

Suppl.Figure 6:
(A) Percentage of GFP+ cells within CD34+ cells prior transplantation was comparable between the groups. (B/C) Human CD34+ cells were transduced with the indicated lentiviruses and injected i.h. into newborn *rag2*$^{-/-}$*γc*$^{-/-}$ mice. 8 weeks later liver, BM and spleen were analysed. Cell suspensions were stained with human CD33 (left) or CD19 (right) and murine CD45 antibodies, and the percentages of GFP+ cells within the human populations were determined. Dot blots of representative BM cells are shown (B). Bars represent percentages of GFP+ cells within the CD33+ or CD19+ population (means +/- SEM from n=7-14 mice, 4-6 independent experiments). Significant p-values (Mann-Whitney-Test) for CD33+ cells: Luci shRNA vs. BIM shRNA: liver p=0.0001, BM p=0.002, spleen p=0.02; Luci shRNA vs. BMF shRNA: liver p=0.001, BM p=0.002, spleen p=0.04. For CD19+ cells: Luci shRNA vs. BIM shRNA: liver p=0.001, BM p=0.02, spleen p=0.0002; Luci shRNA vs. BMF shRNA: liver p=0.001, BM p=0.02, spleen p=0.002 (C).

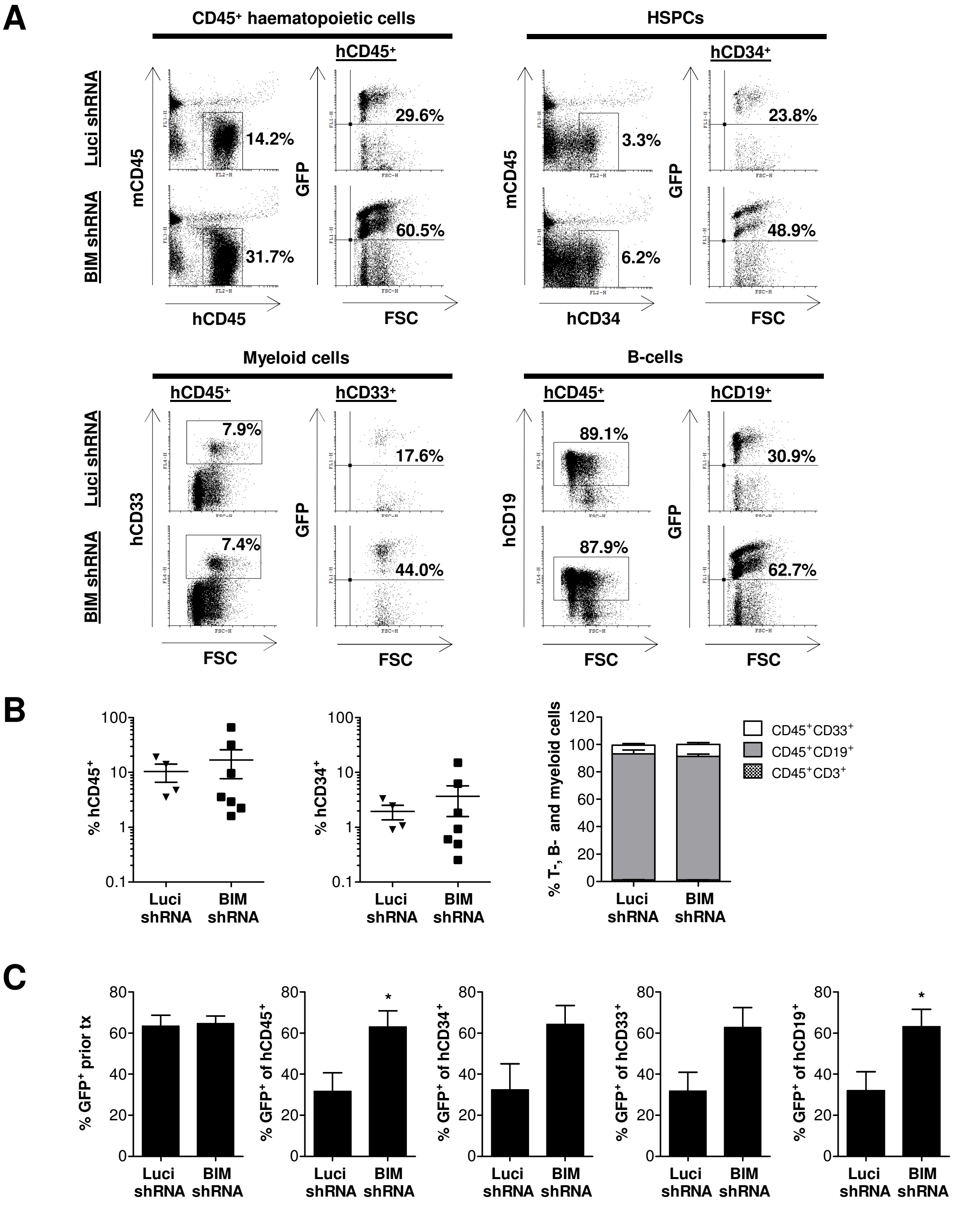

Suppl.Figure 7:
(A-C) 7 weeks old *rag2$^{-/-}$γc$^{-/-}$* mice were transplanted (i.v.) with human CD34^{+} cells transduced with the indicated lentiviruses. 8 weeks later mice were killed and BM was harvested. (A) Representative dot plots of CD45^{+}, CD34^{+}, CD33^{+} and CD19^{+} cell populations. (B) Total BM engraftment of human CD45^{+} cells (left) as well as percentages of immature CD34^{+} cells (middle) and mature cell populations (right) were comparable between the two groups. (C) Percentage of GFP^{+} cells prior transplantation was similar between the groups (left). However, knockdown of BIM increases percentages of GFP^{+} cells in all BM subpopulations analysed. Bars represent means +/- SEM of n= 4-7 mice from 3 independent experiments. Mann-Whitney-Test was used, significant p-values are indicated by asterisks (p=0.04 in CD45^{+} cells; p=0.02 in CD19^{+} cells).
